# Characterization of the mechanism of prolonged adaptation to osmotic stress of *Jeotgalibacillus malaysiensis* via genome and transcriptome sequencing analyses

**DOI:** 10.1038/srep33660

**Published:** 2016-09-19

**Authors:** Amira Suriaty Yaakop, Kok-Gan Chan, Robson Ee, Yan Lue Lim, Siew-Kim Lee, Fazilah Abd Manan, Kian Mau Goh

**Affiliations:** 1 Universiti Teknologi Malaysia, Faculty of Biosciences and Medical Engineering, 81300 Skudai, Johor, Malaysia; 2 University of Malaya, Division of Genetics and Molecular Biology, Institute of Biological Sciences, Faculty of Science, 50603 Kuala Lumpur, Malaysia

## Abstract

*Jeotgalibacillus malaysiensis*, a moderate halophilic bacterium isolated from a pelagic area, can endure higher concentrations of sodium chloride (NaCl) than other *Jeotgalibacillus* type strains. In this study, we therefore chose to sequence and assemble the entire *J. malaysiensis* genome. This is the first report to provide a detailed analysis of the genomic features of *J. malaysiensis*, and to perform genetic comparisons between this microorganism and other halophiles. *J. malaysiensis* encodes a native megaplasmid (pJeoMA), which is greater than 600 kilobases in size, that is absent from other sequenced species of *Jeotgalibacillus*. Subsequently, RNA-Seq-based transcriptome analysis was utilised to examine adaptations of *J. malaysiensis* to osmotic stress. Specifically, the eggNOG (evolutionary genealogy of genes: Non-supervised Orthologous Groups) and KEGG (Kyoto Encyclopaedia of Genes and Genomes) databases were used to elucidate the overall effects of osmotic stress on the organism. Generally, saline stress significantly affected carbohydrate, energy, and amino acid metabolism, as well as fatty acid biosynthesis. Our findings also indicate that *J. malaysiensis* adopted a combination of approaches, including the uptake or synthesis of osmoprotectants, for surviving salt stress. Among these, proline synthesis appeared to be the preferred method for withstanding prolonged osmotic stress in *J. malaysiensis*.

The marine environment contains an abundance of halophilic microorganisms. For these microorganisms, salinity and osmotic stress tolerance are prerequisites for survival. To date, two general adaptive strategies to achieve such tolerance have been reported^1^. First, certain groups of halophiles, such as the extreme halophile *Salinibacter ruber*, maintain favourable osmotic pressure by accumulating high cytoplasmic concentrations of potassium ions (K^+^). This adaptive approach, widely referred to as the ‘salt in’ strategy, requires a specific set of proteins, including (i) influx systems, such as the Ktr, Kdp (KdpFABCDE), Trk (TrkA, TrkE, TkrG and TrkH), and Kup (formerly TrkD) systems, and (ii) passive transport via K^+^ channels and porins (OmpR, and EnvZ)^2^,^3^. For detailed descriptions of the role and regulation of K^+^ in bacteria, readers can refer to the excellent review article published by Epstein^4^. In particular, the extreme halophilic archaeon *Halobacterium salinarum* was shown to take up large amounts of K^+^ to maintain an intracellular K^+^ concentration higher than that of sodium ions (Na^+^) in the environment^3^. In this process, cells accumulate K^+^ while exporting Na^+^ via passive transport via a sodium-potassium ATPase pump^5^. In addition to acting as osmotic solute, K^+^ functions as an activator of intracellular enzymes, a regulator of cytoplasmic pH, and to promote the accumulation of other compatible solutes^4^.

The second osmoadaptation method employed by halophiles is the ‘organic solutes in’ strategy. This strategy is a universal approach utilised by halophilic algae and methanogenic archaea, as well as by halotolerant and halophilic bacteria^1^, including the moderate halophiles *Chromohalobacter salexigens*^6^ and *Halobacillus halophilus*^7^. The osmolytes used by these microorganisms are primarily comprised of sugars (sucrose and trehalose), polyols (glycerol, glucosylglycerol, mannosylglycerol, and arabitol), amino acids (glutamine, proline, alanine, and derivatives), quaternary amines (betaines and choline), or ectoines (ectoine and β-hydroxyectoine)^8^. Notably, the majority of these organic compounds maintain the osmotic balance of the cells without interfering with cellular metabolic pathways^3^,^6^.

To date, many halophiles have been well characterized; however, *Jeotgalibacillus* remains one of the least-studied genera in terms of the number of published reports, as well as the total number of strains characterized. *Jeotgalibacillus* spp. are rod-shaped bacteria that are members of phylum *Firmicutes* (family *Planococcaceae*, order *Bacillales*, and class *Bacilli*). Currently, six species of *Jeotgalibacillus* have been identified: *J. alimentarius*^9^, *J. marinus*^10^, *J. campisalis*^10^, *J. salarius*^10^, *J. soli*^11^, and the most recently characterized type strain, *J. malaysiensis*^12^. Of these, *J. soli* was isolated from soil while the other microorganisms were isolated from salty environments.

Due to the lack of studies of *Jeotgalibacillus* spp., there is little insight into the biology of this genus. In this work, we aimed to further our understanding of these microorganisms, using *J. malaysiensis* as a model. Specifically, we analysed and compared the complete genome sequence of this microorganism, together with draft genomes of *J. alimentarius*, *J. campisalis*, and *J. soli*, to the published genomes of five halophilic bacteria and of one halophilic archaeon. Moreover, we evaluated the transcriptomic responses of *J. malaysiensis* to osmotic stress via RNA sequencing (RNA-Seq) analysis of cells cultivated under low and high NaCl conditions.

## Results

### General genomic information

Comparative genomic analyses were performed between *J. malaysiensis* (JMA) and the halophilic microorganisms *J. alimentarius* (JAL)^13^, *J. campisalis* (JCA)^14^, *J. soli* (JSO)^15^, *Planococcus halocryophilus* (PLA)^16^, *Salinibacter ruber* (SRU)^2^, *Chromohalobacter salexigens* (CHR)^17^, *Halobacillus halophilus* (HAH)^18^, and *Dehalobacter restrictus* (DEH)^19^ (Table 1). The halophilic archaeon *Halobacterium salinarum* (HAL)^20^ was also included in the analysis where appropriate. *J. malaysiensis* was selected as the reference genome, unless otherwise specified. The genome maps for *J. malaysiensis* are provided as Supplementary material (Fig. S1).

**Figure 1:**
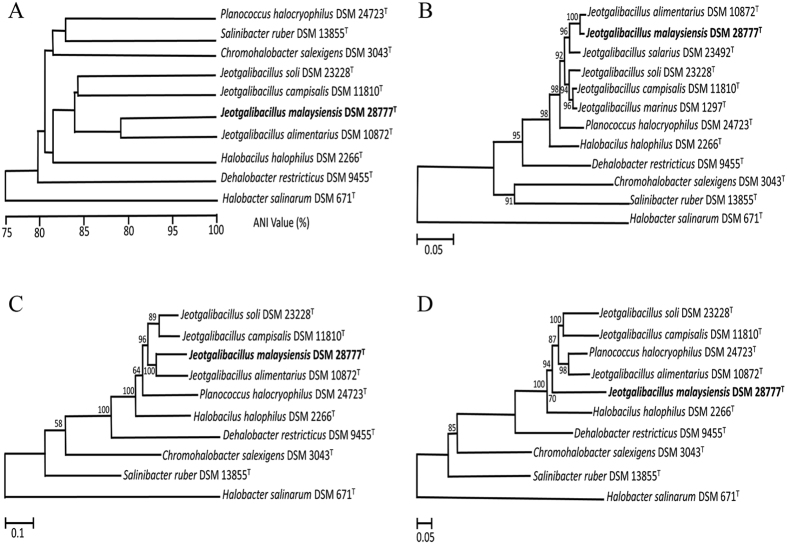
Phylogenetic relationships of the 10 halophiles examined in this study. Phylogenetic trees were constructed based on (**A**) the average nucleotide identity (ANI), as well as the sequence of the (**B**) 16S rRNA, (**C**) *ftsZ*, and (**D**) *dnaA* loci, of each organism via the Neighbor-joining method with 1,000 bootstrap replications. *Halobacterium salinarum* served as the outgroup for all trees.

Phylogenic relationships among strains were evaluated using average nucleotide identity (ANI), as well as sequencing analysis of the 16S rRNA gene and the two housekeeping genes *ftsZ* and *dnaA* (Fig. 1). Phylogenetic tree analyses indicated that all *Jeotgalibacillus* spp. clustered together, but separately from other halophilic microorganisms. Moreover, each of the *Jeotgalibacillus* spp. were identified as unique strains, as the ANI values between strains ranged from 70–80%, which is markedly lower than the species delineation cut-off threshold of 95%^21^. While *Jeotgalibacillus* and *Planococcus*, which are genera of family *Planococcaceae*, exhibited relatively close relationships upon 16S rRNA, *ftsZ*, and *dnaA* phylogenic tree analyses (Fig. 1B–D), ANI analyses indicated that these two genera are actually phylogenetically far apart (Fig. 1A).

Venn diagram analyses showed that there are 1,158 shared orthologous genes among *Jeotgalibacillus* spp., which include coding DNA sequences (CDS) involved in central metabolism, such as genes that play a role in flagellar activity, amino acid transport, translation, ribosomal structure, and biogenesis (Fig. S2A). Analysis of the *J. malaysiensis* genome identified 58 unique CDS, which are predicted to encode proteins such as β-galactosidase, transposase, organic solvent tolerance protein, OstA, and hypothetical proteins. Notably, the gas vacuole operon was present in *J. malaysiensis* but absent from the other *Jeotgalibacillus* spp. Similar operons were found in *P. halocryophilus*, *S. ruber*, *H. salinarum*, and *H. halophilus*, and gas vacuoles are quite common in halophilic bacteria and archaea^22^. Meanwhile, *J. alimentarius*, *J. campisalis*, and *J. soli* possessed 34, 43, and 84 unique CDS, respectively. Detailed information regarding these unique CDS is provided in the Supplementary file (Table S1). Phylogenetically, *J. malaysiensis* was most closely related to *J. alimentarius* (Fig. 1), with these organisms sharing 31 unique CDS that were not present in the other 4 *Jeotgalibacillus* genomes (Table S2).

A comparative analysis of the orthologous genes of the 10 halophilic genomes examined is summarized in the Venn diagram presented in Supplemental Fig. S2B. Notably, among the 292 shared CDS, 85 encode transporters or permeases, while 7 encode proteins related to osmoadaptation and to glycine betaine transporters in particular.

### General metabolism of *J. malaysiensis*

**Figure 2:**
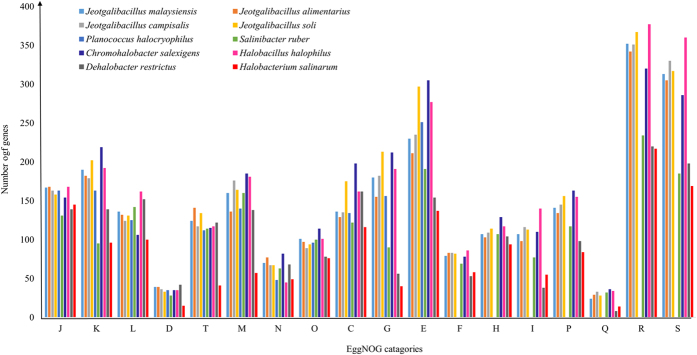
eggNOG (evolutionary genealogy of genes: Non-supervised Orthologous Groups) distribution of the 10 halophilic genomes examined in this study. J: translation, ribosomal structure, and ribosomal biogenesis; K: transcription; L: replication, recombination, and repair; D: cell cycle control, cell division, and chromosome partitioning; O: posttranslational modification, protein turnover, and chaperones; M: cell wall/membrane/envelope biogenesis; N: cell motility; P: inorganic ion transport and metabolism; T: signal transduction mechanisms; C: energy production and conversion; G: carbohydrate transport and metabolism; E: amino acid transport and metabolism; F: nucleotide transport and metabolism; H: coenzyme transport and metabolism; I: lipid transport and metabolism; Q: secondary metabolite biosynthesis, transport, and catabolism; R: general function prediction only; S: function unknown.

**Figure 3:**
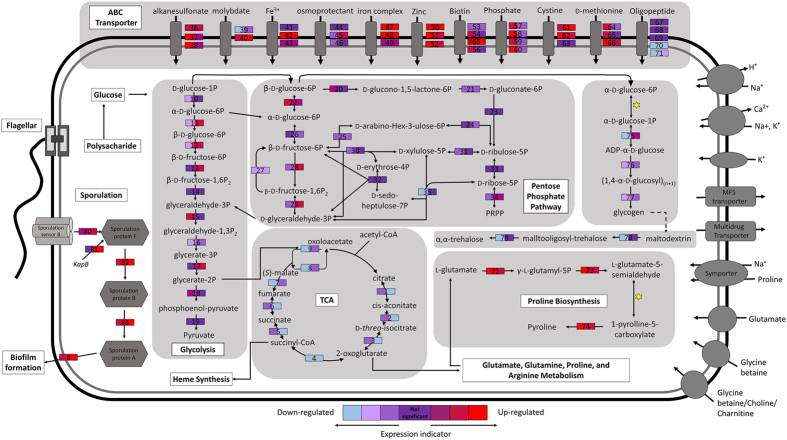
Model of metabolic pathways and important cell components in *Jeotgalibacillus malaysiensis.* Coloured protein boxes indicate expression levels at 10% (left) and 20% (right) NaCl (w/v). *Spontaneous reaction. A list of the enzymes and genes involved in these pathways are provided in Supplemental Table S6, while the list of all up- or downregulated data are provided in Table S7.

Protein-coding genes were functionally categorized using the eggNOG (evolutionary genealogy of genes: Non-supervised Orthologous Groups) Database (Fig. 2). In total, 224 complete metabolic pathways, including the glycolysis, gluconeogenesis, Krebs cycle, tricarboxylic acid (TCA) cycle, and pentose phosphate (PP) pathways, comprising 1,247 enzymatic reactions, were detected in *J. malaysiensis* (Table S3). Thirty-five CDS in *J. malaysiensis* were linked to amino acid and cofactor synthesis pathways, while 44 CDS were assigned to prosthetic group and electron carrier biosynthesis. Based on gene annotation and KEGG (Kyoto Encyclopaedia of Genes and Genomes) Database analyses, a simplified model of the metabolism and important cell components of *J. malaysiensis* is illustrated in Fig. 3.

### Characterization of the *J. malaysiensis* megaplasmid

Four of the strains examined possessed plasmid(s) (Table S4). Four plasmids of varying size were detected in the archaeon *H. salinarum* (Table S4). Notably, while *J. malaysiensis* was found to harbour an extraordinary large 603,070 bp megaplasmid (Fig. S1), designated pJeoMA (Accession number: CP 009417), plasmids were not detected in other *Jeotgalibacillus* spp. using PlasmidFinder-1.3^23^. The existence of pJeoMA in *J. malaysiensis* was confirmed by pulse field gel electrophoresis (PFGE) analysis (Fig. S3). Analysis of this megaplasmid identified 828 CDS, among which 77% were of unknown function. The remaining CDS were predicted to function in DNA replication, DNA methylation, flagella formation, and DNA transfer. Furthermore, pJeoMA contains an additional 40 tRNA loci to the 79 tRNA detected on the *J. malaysiensis* chromosome (Table S5). Indeed, the pJeoMA megaplasmid encodes at least one tRNA locus for each amino acid; however, the reason of this abundance of tRNAs remains unclear. Lastly, the circular megaplasmid contained 24 loci encoding non-intact phage proteins, which could have arisen through horizontal gene transfer events.

### General stress response genes in *J. malaysiensis*

Based on SEED function analysis, 122 CDS in *J. malaysiensis* were predicted to play roles in various stress responses, with the majority of these being associated with osmotic stress (13 CDS), oxidative stress (44 CDS), heat shock (18 CDS), and the regulation of stress response genes (30 CDS). CDS related to the heat shock response included the molecular chaperones DnaJ and DnaK, and the heat shock protein GrpE (JMA_21960−90), which are under the control of an HrcA family transcriptional regulator. *J. malaysiensis* also harbours several CDS predicted to encode gas vesicle proteins: JMA_13400, JMA_24950, JMA_32060, JMA_32080, and JMA_32180. Notably, gas vacuoles have been shown to facilitate the positioning of marine aquatic cells in locations favourable for aerobic respiration. Lastly, there were 17 CDS predicted to encode proteins involved in the synthesis of flavohaemoglobins (flavo Hbs), haemoglobins (Hbs), or truncated haemoglobin (tr Hbs). In general, flavo Hbs detoxify nitric oxide, while Hbs and tr Hbs are responsive proteins expressed during oxygen-limiting conditions. As such, these genes are important for the survival of aerobic bacteria such as *J. malaysiensis*.

### Overview of the osmotic adaptation strategies and transcriptomic responses of *J. malaysiensis*

To characterize the mechanism by which *J. malaysiensis* adapts to osmotic stress, cells cultivated in marine broth (MB) supplemented with 2%, 10%, or 20% (w/v) NaCl were subjected to RNA-Seq and genomic analyses. The seawater samples from which *J. malaysiensis* was isolated contained 12,000 mg/L sodium, 24,535 mg/L chloride, 5,550 mg/L hardness, 2,490 mg/L sulphate, 1,100 mg/L magnesium, 446 mg/L potassium, and other compounds. Meanwhile, the NaCl content in the open sea is generally between 2 and 3.5%^24^. Based on these data, we decided to use cultures grown in the presence of 2% (w/v) NaCl as controls for baseline expression. Accordingly, cultivation in media containing 10% and 20% (w/v) NaCl was used to model an osmotic upshift. The total read counts generated for 2%, 10%, and 20% (w/v) NaCl experiments were 13,271,379, 14,562,786, and 6,653,899. These reads were subsequently mapped to the *J. malaysiensis* genome and megaplasmid. Normalisation was performed using Reads Per Kilobase of transcript per Million mapped reads (RPKM) and Relative Log Expression (RLE), as well as Trimmed Mean of M-values (TMM). Since the coefficient of variation (CV) value for the TMM method (0.5686) was lower than that for RPKM (0.5734) and RLE (0.5702), TMM was utilised for subsequent analyses. A total of 4,130, 4,158, and 4,152 genes were detected in cells cultivated in the presence of 2%, 10%, and 20% (w/v) NaCl, respectively, by RNAseq analysis.

In this work, fold-changes (FC) in gene expression were calculated as “Mean TMM_case_/Mean TMM_control_”, where case corresponds to cell cultivated in 10% or 20% NaCl and control corresponds to cells cultivated in 2% NaCl. Pairwise differentially expressed gene (DEG) analyses were performed by examining the average data from each group. In general, DEG analysis detected genes that were up and down regulated between cells cultivated in 10% or 20% (w/v) NaCl (condition 1 and 2, respectively) vs. 2% (w/v) NaCl (control). DEGs exhibiting at least a two-fold increase in FC values were analysed further. Genes with low magnitude TMM values were not taken into consideration during the analysis. Unless otherwise stated, the DEGs described herein correspond to those located on the *J. malaysiensis* chromosome.

Under 10% (w/v) NaCl conditions, we detected 2,451 DEG. Of these, 1,404 were up-regulated and 1,047 were down-regulated. Meanwhile, 2,380 DEG (1,012 up-regulated and 1,368 down-regulated) were detected under 20% (w/v) NaCl conditions. Compared to the levels of expression detected in the presence of 2% (w/v) NaCl, DnaA was down-regulated when cultured in the presence of both 10% and 20% (w/v) NaCl (FC of 0.79 and 0.24, respectively). In contrast, several general stress-related proteins (JMA_01980, JMA_12870, JMA_25680, and JMA_41330; FC 2.7–10) and at least 10 genes involved in sporulation (FC 2.5–25.8) were up-regulated in response to high salt concentrations. These findings therefore confirm that cultivation in medium containing 10% or 20% (w/v) NaCl resulted in cellular stress.

Genes exhibiting significant changes in expression were also mapped to the KEGG Database. An overview of the DEGs associated with general carbohydrate and energy metabolic pathways are represented in Table 2. Osmotic stress significantly affected carbohydrate, energy, and amino acid metabolism. Specifically, most genes involved in carbohydrate metabolism (i.e., glycolysis, TCA cycle, and butanoate metabolism), which are essential for energy production^25^, were down-regulated under saline stress conditions. For example, fructose-bisphosphate aldolase class 1, fructose-1,6-bisphosphatase 2, and the 2-oxoglutarate dehydrogenase E1 components isocitrate dehydrogenase and 2-oxoglutarate dehydrogenase E1 and E2, which are components of the glycolysis and citrate cycle, respectively, were down-regulated between 0.3–0.5-fold and 0.02–0.2-fold at 10% and 20% (w/v) NaCl, respectively. In contrast, many of the genes involved in the PP pathway were up-regulated in response to salt stress, which is consistent with the previously characterized role of this pathway in protection against oxidants^26^.

At high NaCl concentrations, intracellular reactive oxygen species (ROS) levels are expected to be elevated^27^. The bacterium down-regulates the catalase peroxidase because of the high ROS concentration. As such, it appears that this organism re-routes carbohydrate flux from the glycolysis to the PP pathway to counteract perturbations in the cytoplasmic redox state by increasing cellular levels of the antioxidant cofactor NADPH. Saline stress also appears to lower the rate of carbohydrate metabolism, thereby having a negative effect on fatty acid biosynthesis, particularly at 20% (w/v) NaCl. Indeed, genes involved in fatty acid degradation were markedly down-regulated under both 10% and 20% (w/v) NaCl conditions.

### Physiological changes in *J. malaysiensis* during osmotic stress

During sudden hyperosmotic shock, most non-halophilic cells will undergo plasmolysis, a process resulting in shrinkage of the cytoplasmic volume. To examine the morphology of cells exposed to such prolonged osmotic shock, we analysed *J. malaysiensis* cells grown overnight (18 hours) under 2%, 10%, and 20% (w/v) NaCl conditions by field emission scanning electron microscopy (FESEM). In medium with elevated salt content, cells were slightly smaller and were uneven in shape (data not shown). In addition to the loss of cytoplasmic content, asymmetrical cell shape could be due to down-regulation of gas vesicle proteins and altered expression of the shape-determining proteins MreC and RodA. Under 10% and 20% (w/v) NaCl salt stress, cells also appeared slightly longer than those grown in 2% (w/v) NaCl. The cause for this is not clear, but could be due to endospore production.

**Figure 4:**
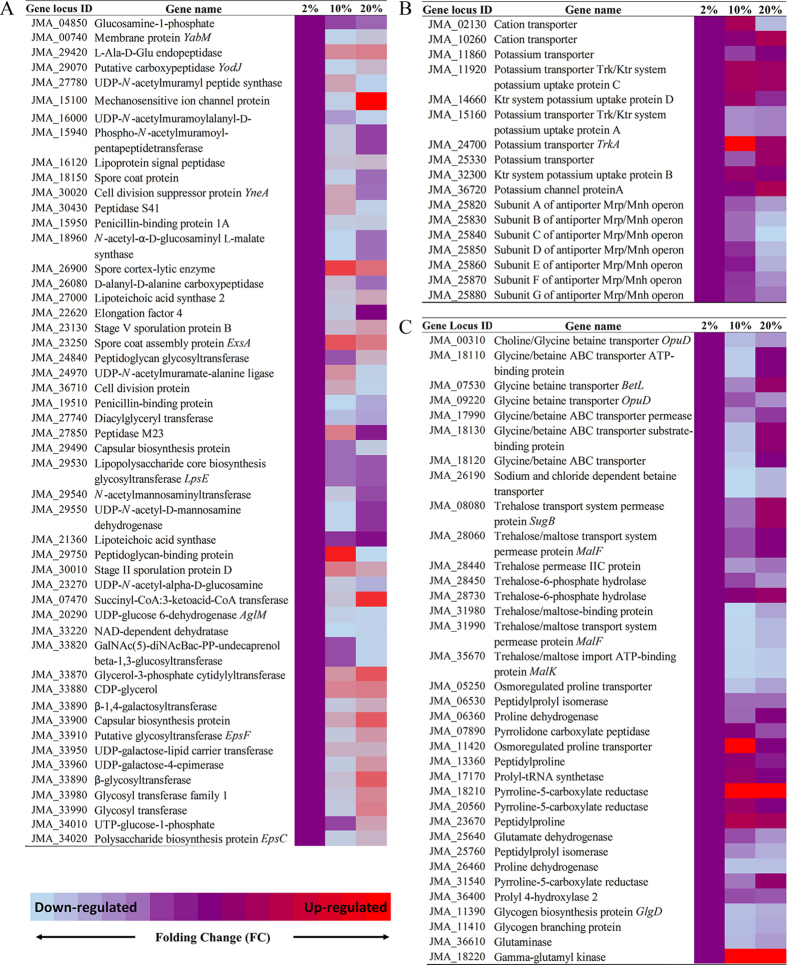
Heat map of the differential expression profiles for (**A**) genes involved in cell wall, membrane, and envelope biosynthesis, (**B**) genes involved in K^+^ uptake and Na^+^ efflux, and (**C**) genes involved in synthesis and uptake of compatible solutes in *Jeotgalibacillus malaysiensis* at 10% and 20% (w/v) NaCl concentrations. Cells cultivated at 2% (w/v) NaCl were used as a control.

The cell wall and cell membrane of *J. malaysiensis* likely underwent compositional changes during osmotic stress, as enzymes involved in cell membrane fatty acid and lipid synthesis were transcriptionally repressed (Fig. 4A). In particular, fatty acid desaturase (JMA_06180) exhibited a 0.6- and 0.1-fold reduction in expression in the presence of 10% and 20% (w/v) NaCl, respectively. Conversely, the mechanosensitive (MS) ion channel protein (JMA_15100) was up-regulated 2.2- and 14.2-fold in media containing 10% and 20% (w/v) NaCl, respectively. Meanwhile, the expression of JMA_36860, a gene predicted to encode another MS channel protein (MscS), was up-regulated in the presence of 10% but not 20% (w/v) NaCl. In addition, many of the 42 DEGs with putative functions associated with flagellar biosynthesis or assembly were down-regulated in response to osmotic stress, suggesting that *J. malaysiensis* inhibits flagellar synthesis under these conditions. Notable exceptions, however, included two genes located on the megaplasmid, flagellin A (JMA_44410) and flagellar filament core protein (JMA_44420), which exhibited 9.5- and 5.6-fold increases in expression in the presence of 10% and 20% (w/v) osmotic stress, respectively. These findings are therefore consistent with an earlier study, which proposed that flagellin production in *H. halophilus* was dependent on chloride concentrations^28^. Lastly, we observed enhanced expression of the gene cluster involved in exopolysaccharide (EPS) and capsular biosynthesis at 20% (w/v) NaCl, which may have been due to the typical production of biofilms that occurs under stressful conditions.

### Role of salt in cytoplasm and osmolytes in *J. malaysiensis*

Generally, *J. malaysiensis* utilizes the TRK system for regulating K^+^ uptake. Figure 4A depicts a gene expression profile heat map for genes involved in K^+^ uptake and Na^+^ efflux during high osmotic stress. Notably, a gene encoding TrkA (JMA_24700) was up-regulated 7-fold at 10% NaCl, but only 2.3-fold at 20% (w/v) NaCl. Meanwhile, Trk potassium uptake proteins C (JMA_11920) and A (JMA_15160) were up- and down-regulated in the presence of high salt concentrations, respectively. Additionally, genes encoding cations transporter (JMA_10260), potassium transporter K^+^/H^+^ antiporter (JMA_25330), potassium channel protein (JMA_36720), and Ktr system potassium transporter B (JMA_32300) were up-regulated in the presence of 10% and/or 20% (w/v) NaCl. The *mnhBCDEF* operon, which contains genes encoding a Na^+^/H^+^ antiporter and plays an important role in the salt stress tolerance of many bacteria^3^, was also detected in the *J. malaysiensis* genome; expression of this operon, however, was down-regulated upon osmotic upshift.

Table 3 summarizes the potential osmolytes that may be used by the 10 selected halophiles during stress, based on genome annotation analysis and/or the results of previous publications. Information for *Bacillus subtilis* (BSU) and *Escherichia coli* (ECO) was also included, as the osmoadaptation strategies of these bacteria have been widely studied^3^,^29^,^30^. Table 4 provides an exhaustive list of putative *J. malaysiensis* genes that are associated with the use of osmoprotectants, including 4 glycine betaine transporters (OpuD and BetL; JMA_00310, JMA_07530, JMA_09220, JMA_17990); 2 CDS encoding a putative osmotically activated L-carnitine/choline ABC transporter (JMA_18120–JMA_18130); 2 L-proline glycine betaine ABC transport system permease proteins (ProV; JMA_09860 and JMA_18110); and 3 choline-sulphatases (EC 3.1.6.6) (JMA_06290, JMA_28560, and JMA_29630), which are predicted to be involved in the first step of the synthesis of choline from choline sulphate. Additionally, all *Jeotgalibacillus* strains encoded multiple glycine betaine transporter genes, which function in transporting betaine into the cytoplasm (Table 4).

Certain halophiles, including those phylogenetically closer to *J. malaysiensis* (i.e., *Salinibacter ruber*, *Chromohalobacter salaxigens*, and *Planococcus halocryophilus*) utilise betaine and choline as osmolytes^6^,^16^,^17^. The presence of genes encoding glycine betaine and choline transporters indicates that these osmoprotectants likely play crucial roles in the survival of *J. malaysiensis* upon osmotic shock. Surprisingly, however, each of these transporter genes was down-regulated or exhibited no significant fold-change in expression when cultivated in the presence of 10% or 20% (w/v) NaCl. The lone exception was the glycine betaine transporter BetL, which was up-regulated 2-fold in the presence of 20% (w/v) NaCl. Previous work reported that glycine betaine and choline are common osmoprotectants of halophiles^1^,^3^,^8^, yet the current RNA-seq analyses do not comply with that observation. Interestingly, the TMM transcript reads for glycine betaine transporters were expressed at high levels, compared to those of ABC transporters not related to osmotic responses, when *J. malaysiensis* was cultivated in media supplemented with 2% (w/v) NaCl. This finding could indicate that glycine betaine/choline transporters are actively expressed at low salt concentrations (2% (w/v) NaCl), but to a lesser extent in the presence of high NaCl concentrations. To confirm the role of glycine betaine in *J. malaysiensis*, the microorganism was cultivated in MB supplemented with 20% (w/v) NaCl with and without glycine betaine (0.01 M). Indeed, under prolonged stress conditions (12 hours), *J. malaysiensis* exhibited slightly enhanced growth in media supplemented with glycine betaine compared with that in cultures lacking artificial supplementation of the osmoprotectant (Fig. S4).

Figure 4C summarizes the expression profiles of genes involved in the usage of other osmolytes. While ectoine is a common osmolyte produced by halophilic bacteria^8^, the ectoine synthesis gene was not detected in the genomes of any *Jeotgalibacillus* strains. In contrast, this gene was found to be encoded by *C. salexigens*^6^ and *H. halophilus*^31^ (Table 3). Meanwhile, the genes responsible for producing osmolytes such as glutamate (GltA, JMA_24240; GltB, JMA_34190; and GltD, JMA_34180), trehalose (GlgA, JMA_11380; GlgB, JMA_11410; GlgP, JMA_11370; GlgC, JMA_11390, JMA_11400, and JMA_11480; and Aamy, JMA_13850), and proline (ProA, JMA_18230; ProB, JMA_18220; and ProC, JMA_18210, JMA_20560, and JMA_31540) were detected in the *J. malaysiensis* genome. Notably, expression of the proline symporter PutP (JMA_11420) was increased 8-fold in the presence 10% NaCl stress, indicating an increase in proline uptake under these conditions, but was down-regulated in the presence of 20% (w/v) NaCl. Proline synthesis genes were also up-regulated in response to the osmotic upshift in *J. malaysiensis*. Moreover, the gene encoding the protein that catalyses the last step in the transformation of glutamate to proline was up-regulated 223-fold in the presence of 10% (w/v) NaCl, but only 23-fold in the presence of 20% (w/v) NaCl, indicating that the microorganism exhibits an increased capacity to biosynthesize proline from L-glutamate in response to salt stress. Consistent with this model, expression of gamma-glutamyl kinase (JMA_18220), which is involved in the production of L-glutamate, was also up-regulated in cells exposed to 20% NaCl. A previous study demonstrated that there is an increase in the cytoplasmic levels of glutamate in most prokaryotes after exposure to high osmolarity^32^. However, the genes predicted to be responsible for glutamate and glutamine synthases in *J. malaysiensis* were down-regulated during prolonged osmotic stress. Indeed, similar to the strategy employed by *H. halophilus*, *J. malaysiensis* appears to preferentially accumulate proline over glutamate and glutamine in response to increasing salt concentrations^31^.

### Functions of inorganic ions transporters during osmotic stress

Saline stress was shown to affect iron homeostasis in *Bacillus* sp. N16-5, *C. salexigens*, and *Helicobacter pylori*^33^,^34^,^35^. In *J. malaysiensis*, hyper salinity (20% NaCl) resulted in a 7-fold increase in Fur-family transcriptional factor (JMA_21570) expression, while cultivation in the presence of 10% (w/v) NaCl yielded a 3.7-fold increase in expression. Furthermore, there was increased expression of all iron ABC transporter permease and ion siderophore proteins (JMA_05930, JMA_14390, JMA_07680, JMA_ 11250, JMA_05590, and JMA_07700) in response to salt stress (FC 7.4–13.8 and 2.2–24.4 at 10% and 20% (w/v) NaCl, respectively). In *C. salexigens*, excess iron leads to increased cytoplasmic concentrations of hydroectoine, as the regulatory protein Fur is also an activator of the ectoine synthesis genes *ectABC*^33^. In addition to excess iron, two general metal ABC transporters (JMA_21580 and JMA_10500) were up-regulated in the presence of high salt concentrations (FC 3.3–6.7). Likewise, expression of the *znuABC* genes, which encode a Zn transporter, was enhanced in response to 10% (w/v) NaCl stress. Conversely, the genes encoding the biotin uptake-related proteins BioY, EcfT, EcfA1, and EcfA2 were down-regulated during osmotic stress. These data suggest that inorganic ions, particularly iron, play a role in osmotic adaptation in *J. malaysiensis*. While the precise role of iron in *J. malaysiensis* has yet to be determined, it could be essential for the redox centres of enzymes involved in the respiratory chain or in intermediary metabolism; however, it is likely not associated with ectoine biosynthesize, as reported elsewhere for *C. salexigens*^33^.

### Protein disaggregation during saline stress

High salt concentrations often result in the denaturation or inactivation of various proteins due to improper folding. Accordingly, molecular chaperones that catalyse the disaggregation of stress-denatured proteins, such as ClpB (JMA_13980) and HSP40 (JMA_10500), a co-chaperone of DnaK, were up-regulated 3.3–6.7-fold in the presence of 10% (w/v) NaCl. Oxidative stress is also associated with osmotic stress^29^. Consistent with this conclusion, salt stress resulted in increased expression of enzymes involved in the repair of oxidized proteins such as JMA_18400, JMA_19120, and JMA_19130. Nevertheless, protein disaggregation is not a perfect solution for misfolded proteins. This likely explains why *degA*, which promotes the expression of degradative protease, was up-regulated in response to osmotic stress. Likewise, the expression of a number of other chaperones and heat/cold shock proteins, including JMA_19060, JMA_21960, JMA_23660, and JMA_23670, was affected by exposure to osmotic stress.

## Discussion

*Jeotgalibacillus* is an underexplored genus of *Planococcaceae*. To gain fundamental knowledge of this genus, we analysed and compared the genome of *J. malaysiensis* to those of other halophiles. *J. malaysiensis* grew efficiently in MB containing 2% (w/v) NaCl, and exhibited similar growth rates in tryptic soy broth (TSB), with or without additional of NaCl. Initially, we intended to use a minimal medium for RNA-Seq experiments. Unfortunately, *J. malaysiensis* grew poorly in media such as R2A and in artificial seawater. Several attempts were made to extract total RNA from cells grown in media lacking additional salt; however, the quality of the resulting RNA was not sufficient for RNA-Seq analysis. Furthermore, while we observed that *J. malaysiensis* can tolerate a maximum NaCl concentration of 30% (w/v), growth at this concentration was dramatically delayed. In contrast, acceptable growth rates were observed at 20% (w/v) NaCl (Fig. S5). Based on these preliminary findings, we chose to cultivate *J. malaysiensis* in MB and to adjust the salinity to 2%, 10%, and 20% (w/v) NaCl to mimic low (2%) and high (10% and 20%) salt concentrations. To evaluate the transcriptomic responses of *J. malaysiensis* under well-defined conditions, however, it will be necessary to utilize a true chemically defined medium.

Collectively, the results of our functional genomic and transcriptomic analyses indicate that *J. malaysiensis* utilizes multiple strategies for adaptation to NaCl stress. The cell wall comprises the initial line of defence against osmotic stress, as the barrier is in direct contact with environment^36^. It is reasonable to assume that hypersaline stress results in modified cell wall, cell membrane, and channel protein composition in *J. malaysiensis*. Consistent with this conclusion, we detected increased expression of MS channel proteins under these conditions, which likely enabled the cells to sense and respond to the osmotic shock. Similar findings were obtained in *B. subtilis*^30^ upon exposure to high salinity conditions. Additionally, saline stress affected the expression of genes that regulate gas vacuoles, chemotaxis, and motility (flagella formation), which could potentially impair cell swarming. At 20% (w/v) NaCl, the observed significant up-regulation of sporulation, EPS production, and capsulation indicates that *J. malaysiensis* initiated biofilm formation in response to the stressful conditions. Meanwhile, the concurrent down-regulation of genes associated with flagellar synthesis was predictable, as this phenomenon was also observed in other genera such as *Rhodobacter* and *Bacillus*^35^,^37^,^38^. The authors of these previous studies suggested that down-regulation of this pathway is needed to reduce Na^+^ ion uptake and to maintain intracellular ion homeostasis^35^. While little is known regarding the relationship between gas vacuole proteins and osmoadaptation, Lee *et al*.^39^ detected enhanced expression of gas vacuole genes in *Streptomyces coelicolor* upon exposure to salt stress, and predicted that gas vesicles may be associated with adaptive stress response^39^. Notably, however, these genes were down-regulated in *J. malaysiensis* under salt stress conditions.

Our data suggest that proline may comprise a critical osmoprotectant for *J. malaysiensis*. During salt stress, aggregated proteins are degraded by proteases into peptides and amino acids. We predict that *J. malaysiensis* recycles these free amino acids, particularly proline and glutamate, and uses them as osmolytes in the presence of hypersalinity stress. Consistent with this conclusion, our RNA-Seq analyses suggested that conversion of glutamate to proline was significantly up-regulated in response to saline stress, while the up-take of proline from the environment was up-regulated during cultivation in the presence of 10% (w/v) NaCl but impaired in the presence of 20% (w/v) NaCl.

The ‘salt in’ strategy of halophiles involves the uptake of K^+^ ions in response to osmotic shock^40^ to maintain homeostatic balance within the cytoplasm. There are 3 prevalent K^+^ uptake systems: KUP, KDP, and TRK^3^. We detected the *trkA* and *trkG* genes within *J. malaysiensis*, indicating that the microorganism employs the TRK system. Indeed, *trkA* was slightly up-regulated in response to elevated NaCl conditions. The KDP system is known as an osmotically inducible system for K^+^ scavenging, when the ion is present at low concentrations^3^; however, this system does not appear to play a significant role in *J. malaysiensis* during prolonged osmotic stress.

For most halophilic bacteria, accumulation of K^+^ is an inadequate strategy to protect against high osmolality^8^. Secondary responses involving the accumulation of compatible solutes are therefore essential for cell survival. *Jeotgalibacillus* spp. are capable of synthesizing glutamate and trehalose via glycogen pathways, as well as importing glycine-betaine. *J. malaysiensis* is also able to acquire choline and proline from the environment. In *Mesorhizobium alhagi*, proline uptake was up-regulated 35.9-fold and trehalose 6-phosphate synthase was up-regulated slightly in response to a salt stress of 2.3% (w/v)^41^. Conversely, in this study, the trehalose permease gene was down-regulated in *J. malaysiensis* at higher NaCl concentrations.

Neither glycine betaine, choline, nor trehalose were added to the medium used for RNA-Seq experiments. Therefore, the concentrations of these compounds in the commercial MB supplemented with 2%, 10%, and 20% (w/v) NaCl were identical. It is conceivable that *J. malaysiensis* requires a greater quantity of osmoprotectants in media with higher salinity [i.e., 20% (w/v) NaCl] than in media with lower salt concentrations. If so, the availability of osmoprotectants within the medium at the point at which total RNA was harvested (12 hours after osmotic shock) would have been limited. Consistent with this conclusion, we observed a reduction in the transcripts for these transporters in the cells grown in media containing 10% and 20% (w/v) NaCl.

Ectoine can be considered a universal marker for halophilic bacteria, as production of this compound is one of the common osmoadaptation strategies of these microorganisms. However, ectoine has also been shown to place a burden on central metabolism biosynthesis in *C. salexigen*^6^. *J. malaysiensis* lacks the *ectABC* genes, indicating that it is incapable of *de novo* ectoine synthesis. Several of the reference halophiles examined in this study are able to synthesis betaine (Table 3); however, the *Jeotgalibacillus* spp. identified to date lack this ability. Pittelkow and Bremer^42^ observed that halophilic microorganism that produce ectoine or a combination of ectoine and proline are more salt tolerant than those that, like *Jeotgalibacillus* spp., rely on glutamate or proline alone. Most halophiles (Table 3) employ multiple osmolyte strategies, including uptake from the environment and biosynthesis. In a previous study, *H. halophilus* was found to produce distinct osmolytes at different NaCl concentrations; for instance, the cells switched from glutamate to proline at high NaCl concentrations^31^.

The detailed *in-situ* response of *J. malaysiensis* within the open ocean remains unclear and may be far more complicated than is currently recognized, as the composition of the solutes available in exogenous sources likely fluctuates over time. The transcriptomic analyses performed here were designed to evaluate the effects of prolonged stress conditions (12 hours) on this organism. Our experimental setup may therefore mimic situations where *J. malaysiensis* cells are flushed from the ocean and trapped in intertidal zones (i.e., salt-marshes), resulting in exposure to profound changes in saline concentrations between tidal inundations. The current RNA-Seq data do not necessarily contradict or support earlier findings, but rather provide insight into the transcriptional responses of *J. malaysiensis* to prolonged stress. We assume that the periodic responses of *J. malaysiensis* to sudden increases or decreases in saline concentration may appear slightly different than the data presented here. As such, monitoring the responses to sudden upshifts in saline stress via transcriptomic, radiolabeling, biochemical, and real-time analyses will provide an overview of the temporal osmolyte-switching strategy employed by *J. malaysiensis*.

## Conclusion

In this study, we performed genomic and transcriptomic analyses of *J. malaysiensis*, and compared the genome of this microorganism to those of other halophilic microorganism. *J. malaysiensis* appears to establish halotolerance via a global cooperation mechanism, rather than by one single approach. During osmotic stress, the physiology and general metabolism of *J. malaysiensis* cells are altered, likely due to the slower growth rate, and the organism appears to preferentially use proline as an osmoprotectant during prolonged osmotic stress. Despite the comprehensive work conducted here, certain inquiries remain unexplained. Specifically, (i) the actual biological role of the pJeoMA megaplasmid in *J. malaysiensis* physiology, (ii) the function of hypothetical proteins that were differentially expressed during osmotic stress, and (iii) the transcriptomic response of *J. malaysiensis* in response to sudden stress have yet to be fully elucidated. The authors hope that the comparative genomic and transcriptomic analyses of *J. malaysiensis* performed here will inspire the scientific community to explore this genus via more in-depth studies in the near future.

## Materials and Methods

### DNA extraction and analysis of general genomic features

Strains *J. alimentarius* DSM 18867^T^, *J. soli* DSM 23228^T^, *J. campisalis* DSM 18983^T^, and *J. salarius* DSM 23492^T^ were obtained from DSMZ (Braunschweig, Germany). The genomic DNA of each strain was extracted using a DNeasy Blood & Tissue Kit (Qiagen, Venlo, Netherlands). The draft genomes of *J. alimentarius*, *J. campisalis*, *J. soli*, and *J. salarius* were sequenced using an Illumina MiSeq system (Illumina, Inc., San Diego, CA, USA), and *de novo* assembly was performed using SPAdes software^43^. Notably, assembly of the sequencing reads obtained from *J. salarius* yielded greater than a thousand short contigs, despite two separate MiSeq sequencing reactions being performed. As a result, genomic information for *J. salarius* was not included in this report. The complete genome of *J. malaysiensis* D5^T^ was sequenced using a PacBio RS II platform (Pacific Biosciences, Menlo Park, CA, USA) and a 10-kb SMRTbell library. *De novo* assembly was performed with the Hierarchical Genome Assembly Process (HGAP) algorithm in the SMRT Portal (version 2.1.1). Protein-coding sequences were predicted by Glimmer software version 3.0^44^ and annotated using BLAST searches of non-redundant protein sequences from the NCBI, Swiss-Prot, NCBI Refseq, COG^45^, IMG-er, and SEED databases. Ribosomal RNA genes were detected using RNAmmer software version 1.2^46^ and transfer RNA genes were detected using tRNAscan-SE software^47^. All genomic data are available at DDBJ/EMBL/GenBank under the accession numbers for *J. malaysiensis* D5^T^ (CP009416), *J. alimentarius* (JXRQ00000000), *J. campisalis* (JXRR00000000), and *J. soli* (JXRP00000000), respectively.

### Water Analysis

Seawater was collected in sterile bottles from Desaru Beach in Johor Bharu, Malaysia, and stored as described earlier by Chan *et al*.^48^. Water analyses were conducted by Allied Chemists Laboratory Sdn. Bhd (Malaysia), following the guidelines of the Public Health Association (APHA) and United States Environmental Protection Agency (USEPA).

### Comparative genomics

The genome sequences of *J. malaysiensis*^49^, *J. alimentarius*^13^, *J. campisalis*^14^, and *J. soli*^15^ were compared to the published sequences of the halophiles *P. halocryophilus*^16^, *S. ruber*^2^, *C. salexigens*^17^, *H. halophilus*^18^, and *D. restrictus*^19^. The genome sequences of these strains were obtained and downloaded from EzGenome (http://ezgenome.ezbiocloud.net/ezg_browse), and the core genome shared by *Jeotgalibacillus* spp. and the six halophiles was analysed and displayed using the CLgenomics program (ChunLab, South Korea). Sequence alignments were performed using ClustalW 2.0 software, while neighbour-joining trees and amino acid compositions were constructed and calculated using MEGA6 software^50^. The general metabolic pathways of each strain were analysed using the KEGG Database and MetaCyc Database^51^,^52^. A 4-way Venn diagram (cut-off value of 70% for orthologous genes) was constructed for *Jeotgalibacillus* spp. using online tools (http://bioinformatics.psb.ugent.be/webtools/Venn/). Furthermore, a 10-way Venn diagram (cut-off value of 25%) was constructed for all genomes manually using COG annotations from the same site.

### Transcriptome sequencing

*J. malaysiensis* was cultivated in quadruplicate in liquid marine broth (MB) containing low [2% (w/v)] and high concentrations [10% and 20% (w/v)] of NaCl for 12 hours. Total RNA was extracted from each sample (mean RIN value = 8.1) using an RNeasy Mini Kit (Qiagen) and further purified using the Qiagen RNase-Free DNase Set, in accordance with the manufacturer’s instructions. Libraries for Illumina sequencing were generated using a TruSeq Stranded mRNA sample prep kit, according to the manufacturer’s protocol. Single-end 50 bp RNA sequencing was then conducted on an Illumina HiSeq 2500 platform at the Korean ChunLab service provider (Seoul, South Korea). Quality-filtered reads were aligned to the *J. malaysiensis* genome using Bowtie2 software^53^. Normalization was performed using RPKM, RLE, and TMM approaches, and the method with the lowest coefficient of variation was selected. The eggNOG Database was used to cluster genes into functionally related groups, while the KEGG Database was used to analyse metabolic pathways^54^. In addition, pathway enrichment analyses using the KEGG Database were performed to identify DEGs that exhibited significant changes in expression, with false discovery rate FDR-corrected P-values ≤ 0.05 and enrichment with Fisher exact test P-values ≤ 0.05. Visualization of the mapping results, DEG analyses, eggNOG, and KEGG were performed using the CLRNASeq program (ChunLab). P-values were designated by the R package’s DESeq2 program, an upgraded version of the DEGSeq algorithm^55^. Raw data have been submitted to the NCBI Sequence Read Archive (SRA) under accession number SRP069110.

## Additional Information

**How to cite this article**: Yaakop, A. S. *et al*. Characterization of the mechanism of prolonged adaptation to osmotic stress of *Jeotgalibacillus malaysiensis* via genome and transcriptome sequencing analyses. *Sci. Rep.* **6**, 33660; doi: 10.1038/srep33660 (2016).

## Supplementary Material

Supplementary Information

## Figures and Tables

**Table 1:** Genomic features of the 10 selected halophilic microorganisms.

	JMA	JAL	JSO	JCA	PLA*	SRU*	CHR*	HAH*	DEH*	HAL*
Strain	DSM28777	DSM18867	DSM18983	DSM23228	DSM24723	DSM13855	DSM3043	DSM2266	DSM 9455	DSM 671
Accession no.	CP009416	JXRQ00000000	JXRR00000000	JXRP00000000	CP009129	CP000159	AAHZ01000000	NC017668	CP007033	NC002607
Status	Complete	Draft	Draft	Draft	Complete	Complete	Complete	Complete	Complete	Complete
Size (bp)	3,516,439	3,364,745	3,650,490	3,776,953	3,196,500	3,551,823	3,696,649	4,150,632	2,943,336	2,000,962
G+C	42.37	43.13	41.06	39.73	39.33	66.13	63.91	41.82	44.56	65.71
CDS	4,444	3,536	3,729	3,938	3,089	2,833	3,298	4,126	2,790	2,749
rRNA	27	7	6	5	24	3	15	21	12	3
tRNA	119	74	69	78	60	44	69	67	52	47
Contigs	2	32	24	24	0	2	1	3	1	5
Gram staining	+	v	v	+	+	−	−	+	−	−
Plasmid	1	0	0	0	1	1	0	2	0	4
Contigs	2	32	24	24	2	2	1	3	1	5
NaCl range (%)	0−30	0−20	0−15	0−9	0−19	11–30	1−25	2−20	Not reported	15−30
Temp range (ºC)	4−50	10−40	4−39	15−40	−10−37	10−50	15−45	15−40	10−37	20−40
Gene Count	4,536	3,557	3,665	3,798	3,217	3,073	3,407	4,224	2,908	2,801
CRISPR	2	0	0	0	0	0	2	0	0	0
W/ Func Pred	3,094	2,796	2,938	3,040	2,531	2,280	2,980	3,216	2,170	1,499
Paralogs	3,048	2,401	2,455	2,742	2,010	2,377	2,681	1,501	1,174	1,441
COG	2,542	2,308	2,489	2,530	1,952	1,809	2,719	2,649	1,758	1,663
KOG	701	656	691	737	581	630	765	793	429	506
Pfam	3,191	2,891	3,022	3,132	2,514	2m343	3,031	3,270	2,296	1,884
TIGRfam	1,211	1,128	1,161	1,210	943	917	1,310	1,264	997	617
IMG Pathway	153	150	160	155	124	202	340	176	129	240
Signal Peptide	161	132	125	130	164	289	284	221	84	51
Transmembrane	1,114	962	998	895	783	895	827	1,144	755	584
Biosynthetic Cluster Gene	236	102	355	143	126	174	131	221	66	78
InterPro	2,055	1843	1,890	2001	1,604	1,525	1,962	3,270	2,311	1,967
Horizontally transferred	180	44	39	55	27	154	252	0	176	33

JMA, *Jeotgalibacillus malaysiensis*; JAL, *Jeotgalibacillus alimentarius*; JCA, *Jeotgalibacillus campisalis*; JSO, *Jeotgalibacillus soli*; PLA, *Planococcus halocryophilus*; SRU, *Salinibacter ruber*; CRO, *Chromohalobacter salexigens*; HAH, *Halobacillus halophilus*; DEH, *Dehalobacter restrictus*; HAL, *Halobacterium salinarum*.

^*^Genomic data were obtained from EzTaxon and IMG-er database.

**Table 2:** Overview of the differentially expressed genes (DEG) involved in carbohydrate and energy metabolism pathways (KEGG) in *Jeotgalibacillus malaysiensis*.

KEGG pathways	Gene name	Gene locus ID	10%	20%
Carbohydrate Metabolism				
Glycolysis/Gluconeogenesis	glucose-specific IIC component	JMA_31330	↑	NS
	aldose 1-epimerase	JMA_30260	↓	NS
	fructose-1,6-bisphosphatase II	JMA_29120	↓	↓
	phosphoenolpyruvate carboxykinase	JMA_25360	NS	↓
	S-(hydroxymethyl)glutathione dehydrogenase	JMA_32360	NS	↓
	fructose-bisphosphate aldolase, class I	JMA_35110	↓	NS
TCA cycle	pyruvate dehydrogenase E1 component α subunit	JMA_09020	↓	↓
		JMA_15220	↓	↓
	isocitrate dehydrogenase	JMA_24230	NS	↓
	malate dehydrogenase	JMA_24240	NS	↓
	succinyl-CoA synthetase α subunit	JMA_16660	NS	↓
		JMA_16670	NS	↓
	2-oxoglutarate dehydrogenase E2 component	JMA_18730	↓	↓
	succinate dehydrogenase/fumarate reductase	JMA_23760	↓	NS
		JMA_23770	↓	↓
		JMA_23780	↓	↓
	2-oxoglutarate dehydrogenase E1 component	JMA_18800	↓	↓
Pentose Phospahte Pathway	glucose-6-phosphate 1-dehydrogenase	JMA_20600	↑	NS
	glucose-6-phosphate isomerase	JMA_25610	↑	NS
	phosphopentomutase	JMA_20380	NS	↑
	ribokinase	JMA_35500	NS	↑
	ribose 5-phosphate isomerase A	JMA_29110	NS	↑
Fructose mannose metabolism	ribose 5-phosphate isomerase B	JMA_30200	NS	↑
	PTS system, glucitol/sorbitol-specific IIC component	JMA_08980	↓	↓
	fructose-1,6-bisphosphatase II	JMA_30420	↓	↓
Pyuruvate	acetate kinase	JMA_24470	↑	NS
	phosphate acetyltransferase	JMA_22990	↑	NS
	acetyl-CoA C-acetyltransferase	JMA_29520	↓	NS
	pyruvate dehydrogenase E1 component alpha subunit	JMA_08600	↓	↓
		JMA_18220	↓	↓
	acetyl-CoA synthetase	JMA_24850	↓	NS
	fumarate hydratase, class II	JMA_13220	↓	NS
	malate dehydrogenase (quinone)	JMA_35120	↓	NS
	malate dehydrogenase	JMA_24220	↓	↓
	2-isopropylmalate synthase	JMA_15400	↓	↓
	acetyl-CoA carboxylase carboxyl transferase subunit alpha	JMA_21030	↓	↓
Propanoate Metabolism	acetate kinase	JMA_24560	↑	↑
	phosphate acetyltransferase	JMA_31050	↑	↑
	acyl-CoA dehydrogenase	JMA_30570	↓	↓
	methylmalonyl-CoA mutase	JMA_20750	↓	↓
		JMA_30460	↓	↓
	2-methylcitrate synthase	JMA_02380	↓	NS
	acetyl-CoA carboxylase carboxyl transferase subunit alpha	JMA_21030	↓	↓
	NADP-dependent alcohol dehydrogenase	JMA_25200	NS	↑
	acetyl-CoA C-acetyltransferase	JMA_30590	↓	↓
	succinyl-CoA synthetase alpha subunit	JMA_16660	↓	↓
		JMA_16670	↓	↓
Butanoate metabolism	butyryl-CoA dehydrogenase	JMA_21680	↓	↓
	3-hydroxybutyryl-CoA dehydrogenase	JMA_30580	↓	↓
	acetyl-CoA C-acetyltransferase	JMA_30590	↓	↓
	3-oxoacid CoA-transferase	JMA_20470	↓	↓
	acyl-CoA dehydrogenase	JMA_30570	↓	↓
Energy Metabolism				
Carbon fixation pathways in carbohydrates	acetate kinase	JMA_24470	↑	↑
	phosphate acetyltransferase	JMA_31050	↑	↑
	acetyl-CoA synthetase	JMA_24850	↓	↓
	acetyl-CoA C-acetyltransferase	JMA_30590	↓	↓
	methylmalonyl-CoA mutase	JMA_20750	↓	↓
		JMA_30540	↓	↓
Fatty acid degradadtion	acyl-CoA dehydrogenase	JMA_30570	↓	↓
	butyryl-CoA dehydrogenase	JMA_21680	↓	↓
	glutaryl-CoA dehydrogenase	JMA_08950	↓	↓
	enoyl-CoA hydratase	JMA_23850	↓	↓
	acetyl-CoA acyltransferase	JMA_26430	↓	↓
	acetyl-CoA C-acetyltransferase	JMA_30590	↓	↓
	long-chain acyl-CoA synthetase	JMA_13570	NS	↓

Up-regulation of a given enzyme is indicated by ↑, while down-regulation is indicated by ↓.

NS = not significant.

**Table 3:** Major osmolyte uptake/synthesis genes present in the sequenced genomes of the 10 halophilic bacteria examined in this study.

Bacteria		JMA	JAL	JCA	JSO	PLA	SRU	CHR	HAH	DEH	HAL	BSU	ECO
Osmolytes													
Glycine Betaine	Synthesis	X	X	X	X	X	X	√	X	X	X	√	√
	Uptake	√	√	√	√	√	√	√	X	X	√	√	√
Choline	Synthesis	X	X	X	X	X	X	X	X	X	X	X	X
	Uptake	√	√	√	√	√	√	√	X	X	X	√	√
Ectoine	Synthesis	X	X	X	X	X	X	√	√	X	X	X	X
	Uptake	X	X	X	√	X	X	√	√	√	X	√	√
Carnitine	Synthesis	X	X	X	X	X	X	X	X	X	X	X	X
	Uptake	√	X	√	X	X	X	√	X	X	X	√	X
Trehalose	Synthesis	√	X	X	X	X	X	√	X	X	X	X	√
	Uptake	√	X	X	X	X	X	X	X	X	X	X	√
Proline	Synthesis	√	X	X	X	√	X	X	√	√	X	√	√
	Uptake	√	√	X	√	√	√	√	√	√	X	√	√
Glutamate	Synthesis	√	X	X	X	X	√	√	√	X	√	X	√
	Uptake	√	√	√	√	X	√	√	√	X	√	√	√

JMA, *Jeotgalibacillus malaysiensis*; JAL, *Jeotgalibacillus alimentarius*; JCA, *Jeotgalibacillus campisalis*; JSO, *Jeotgalibacillus soli*; PLA, *Planococcus halocryophilus*; SRU, *Salinibacter ruber*; CRO, *Chromohalobacter salexigens*; HAH, *Halobacillus halophilus*; DEH, *Dehalobacter restrictus*; HAL, *Halobacterium salinarum*; BSU, *Bacillus subtilis*; ECO, *Escherichia coli*.

**Table 4:** List of putative genes associated with osmolytes in the *Jeotgalibacillus malaysiensis* genome.

Seed Function	COG Gene	COG ID	Gene locus ID
Betaine Glycine/Charnitine			
Glycine betaine transporter OpuD	BetT	COG1292	JMA_00310
Glycine betaine transporter OpuD	BetT	COG1292	JMA_07530
Glycine betaine transporter OpuD	BetT	COG1292	JMA_09220
HTH-type transcriptional regulator BetI	AcrR	COG1309	JMA_34480
L-proline glycine betaine ABC transport system permease protein ProV	TauB	COG1116	JMA_09860
L-proline glycine betaine ABC transport system permease protein ProV	OpuBA	COG1125	JMA_18110
Osmotically activated L-carnitine/choline ABC transporter, permease protein OpuCD	OpuBB	COG1174	JMA_18120
Sodium/glycine symporter GlyP	AlsT	COG1115	JMA_12310
Sodium/glycine symporter GlyP	AlsT	COG1115	JMA_12360
Sodium/glycine symporter GlyP	AlsT	COG1115	JMA_18170
Glycine betaine transporter OpuD	BetT	COG1292	JMA_17990
Osmotically activated L-carnitine/choline ABC transporter, permease protein OpuCD	OpuBC	COG1732	JMA_18130
Glycine dehydrogenase [decarboxylating] (glycine cleavage system P2 protein)	GcvP	COG1003	JMA_21180
Glycine dehydrogenase [decarboxylating] (glycine cleavage system P1 protein)	GcvP	COG0403	JMA_21190
Aminomethyltransferase (glycine cleavage system T protein)	GcvT	COG0404	JMA_21200
Glycine cleavage system H protein	GcvH	COG0509	JMA_26380
DgcB Dimethylglycine demethylase subunit B	GlpC	COG0247	JMA_30600
Glycine oxidase ThiO	DadA	COG0665	JMA_36350
Choline			
Choline-sulfatase	MdoB	COG1368	JMA_06290
Choline-sulfatase	MdoB	COG1368	JMA_28560
Choline-sulfatase	MdoB	COG1368	JMA_29630
Proline			
Threonyl-tRNA synthetase	ProS	COG0442	JMA_17170
Pyrroline-5-carboxylate reductase	ProC	COG0345	JMA_18210
Glutamate 5-kinase	ProB	COG0263	JMA_18220
Gamma-glutamyl phosphate reductase	ProA	COG0014	JMA_18230
Pyrroline-5-carboxylate reductase	ProC	COG0345	JMA_20560
Pyrroline-5-carboxylate reductase	ProC	COG0345	JMA_31540
Glutamate			
Sodium/glutamate symport protein	GltP	COG1301	JMA_04020
Sodium/glutamate symport protein	GltP	COG1301	JMA_08140
Sodium/glutamate symport protein	GltP	COG1301	JMA_13140
Citrate synthase (si)	GltA	COG0372	JMA_24240
Glutamate synthase [NADPH] small chain	GltD	COG0493	JMA_34180
Glutamate synthase [NADPH] large chain	GltB	COG0069	JMA_34190
Trehalose			
PTS system, glucose-specific IIA component	PtsG	COG1263	JMA_11100
1,4-alpha-glucan (glycogen) branching enzyme, GH-13-type	GlgB	COG0296	JMA_11410
PTS system, glucose-specific IIA component	NagE	COG2190	JMA_19110
PTS system, trehalose-specific IIB component	PtsG	COG1263	JMA_28440
Trehalose-6-phosphate hydrolase	AmyA	COG0366	JMA_28450
Trehalose operon transcriptional repressor	PhnF	COG2188	JMA_28490
PTS system, maltose and glucose-specific IIB component	PtsG	COG1263	JMA_31330
Trehalose utilization protein	ThuA	COG4813	JMA_32010
