# Supplementary material for: Characterization of the mechanism of prolonged adaptation to osmotic stress of Jeotgalibacillus malaysiensis via genome and transcriptome sequencing analyses
Source: Sci Rep. 2016 Sep 19;6:33660. doi: 10.1038/srep33660 (PMC5027565; doi:10.1038/srep33660)
Supplement: Supplementary Information [file srep33660-s1.pdf]

Characterization of the mechanism of prolonged adaptation to osmotic stress of *Jeotgalibacillus malaysiensis* via genome and transcriptome sequencing analyses

Amira Suriaty Yaakop[1], Kok-Gan Chan[2], Robson Ee[2], Yan Lue Lim[2], Siew-Kim Lee[2], Fazilah Abd Manan[1], Kian Mau Goh[1*]

[1]Universiti Teknologi Malaysia, Faculty of Biosciences and Medical Engineering, 81300 Skudai, Johor, Malaysia.

[2]University of Malaya, Division of Genetics and Molecular Biology, Institute of Biological Sciences, Faculty of Science, 50603 Kuala Lumpur, Malaysia.

*Corresponding: gohkianmau@utm.my; phone: +607 5557556; fax: +607 5531279

Supplemental Figures

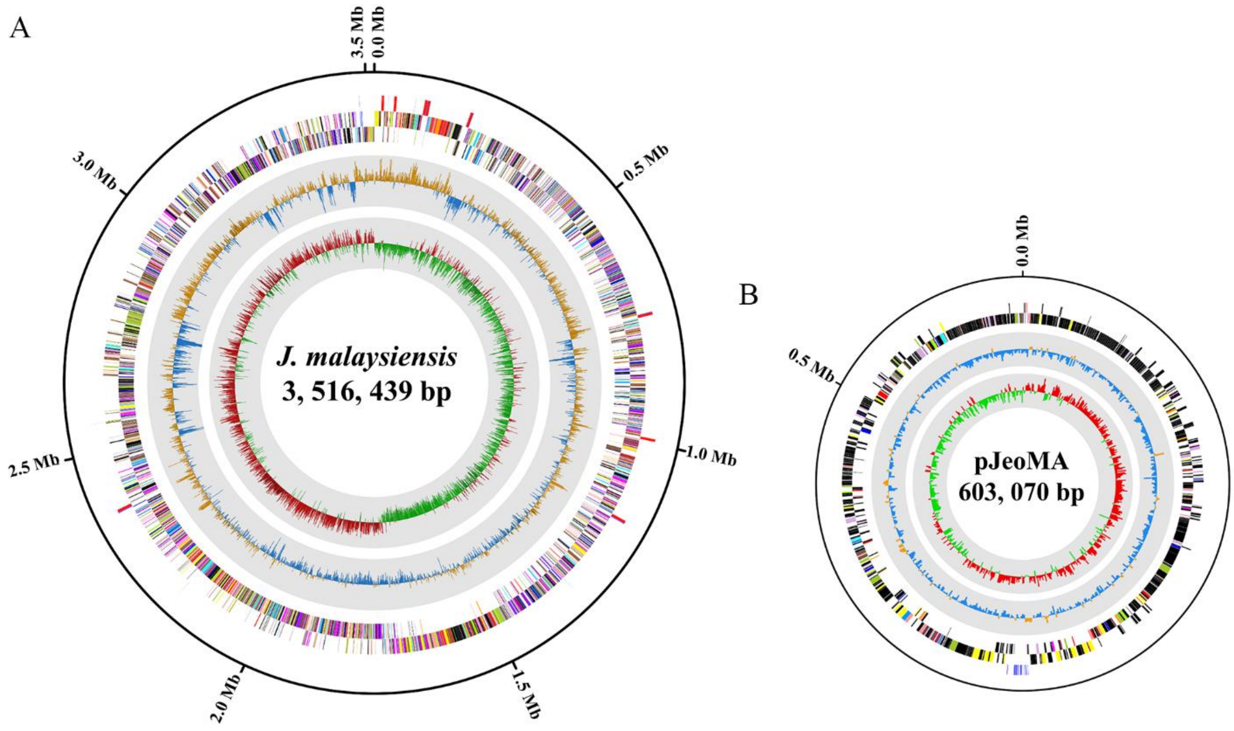

Figure S1. Genome map of the *Jeotgalibacillus malaysiensis* genome and megaplasmid.

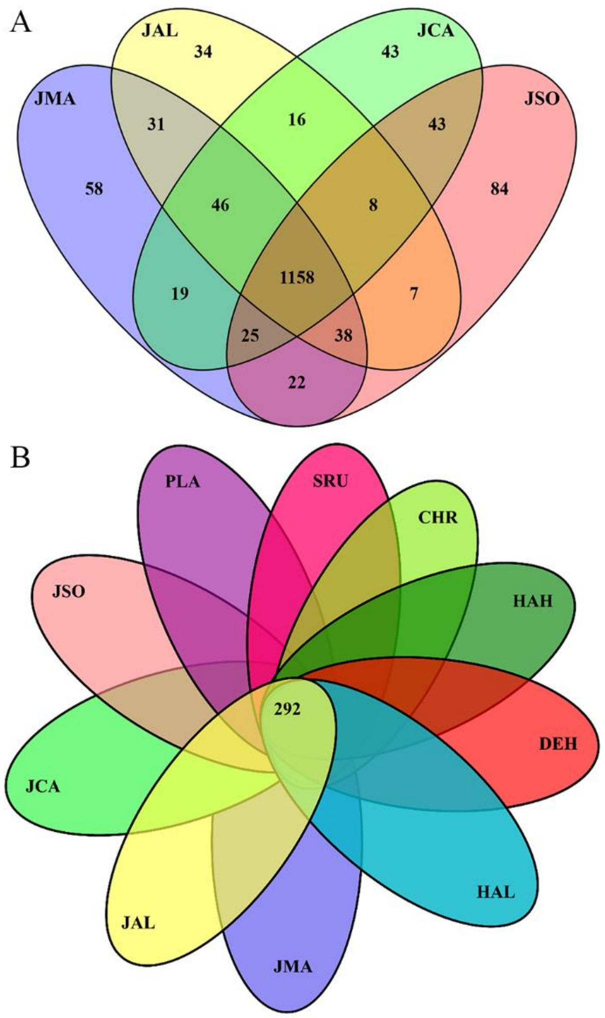

Figure S2. Venn diagram of the orthologous gene shared between (A) *Jeotgalibacillus* spp. and (B) the 10 selected halophilic microorganisms. *Jeotgalibacillus malaysiensis*. JMA, *J. malaysiensis*; JAL, *J. alimentarius*; JCA, *J. campisalis*; JSO, *J. soli*; PLA, *Planococcus halocryophilus*; SRU, *Salinibacter ruber*; CRO, *Chromohalobacter salexigens*; HAH, *Halobacillus halophilus*; DEH, *Dehalobacter restrictus*; HAL, *Halobacterium salinarum.*

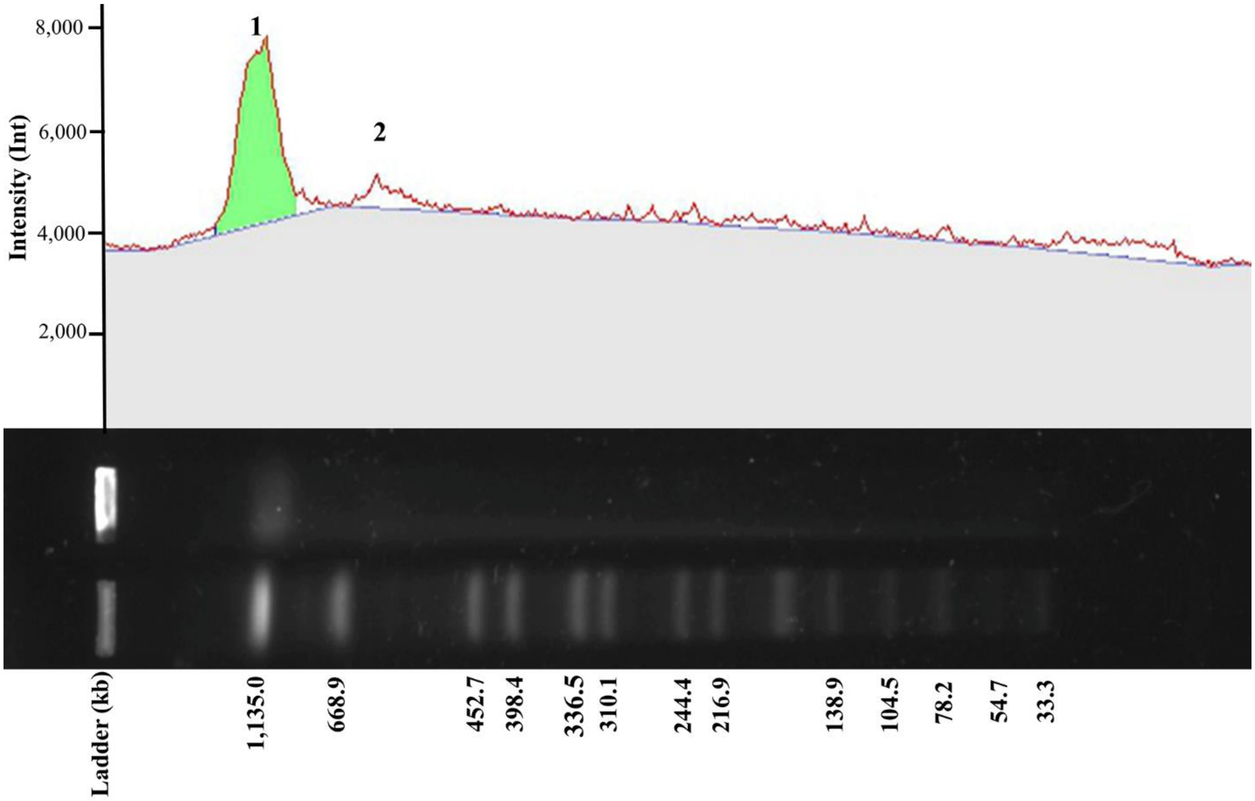

Figure S3. Pulse field gel electrophoresis (PFGE) image of the *Jeotgalibacillus malaysiensis* megaplasmid.

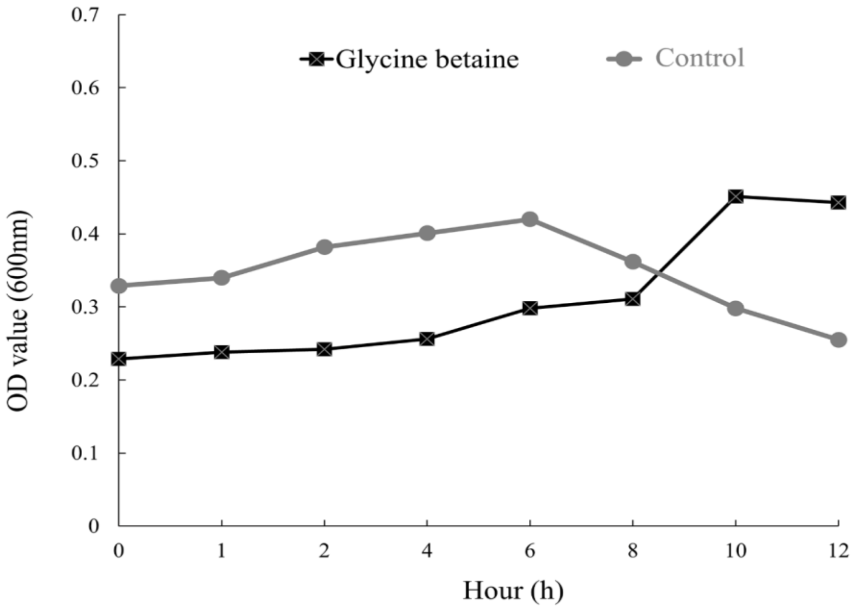

Figure S4. Growth curves of *Jeotgalibacillus malaysiensis* when cultivated in marine broth supplemented with 0.01M of glycine betaine at 20% (w/v) NaCl concentrations.

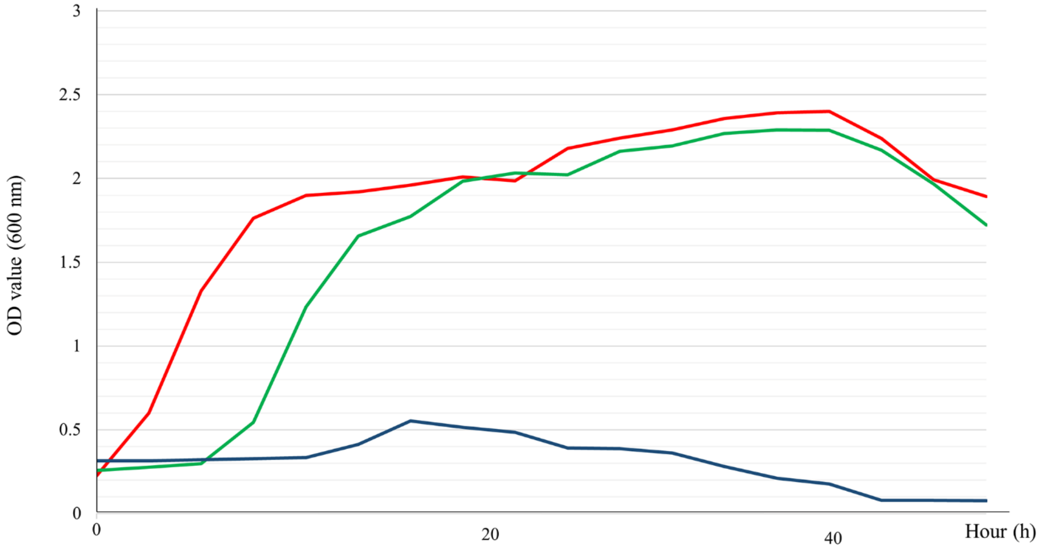

Figure S5. Growth curves of *Jeotgalibacillus malaysiensis* when cultivated in marine broth supplemented with 2% (red), 10% (green), and 20% (blue) NaCl.

Supplemental Tables

Table S1. Coding DNA sequences (CDS) of *Jeotgalibacillus malaysiensis* that are unique compared to other *Jeotgalibacillus* spp. genomes.

***J. malaysiensis* (58 CDS)**	***J. alimentarius* (34 CDS)**	***J. campisalis* (43 CDS)**	***J. soli* (84 CDS)**
Aminoglycoside phosphotransferase	ABC-type antimicrobial peptide transport system, permease component	4-amino-4-deoxy-L-arabinose transferase and related glycosyltransferases of PMT family	2-keto-4-pentenoate hydratase
ATP-utilizing enzymes of ATP-grasp superfamily (probably carboligases)	ABC-type xylose transport system, periplasmic component	ABC-type branched-chain amino acid transport system, permease component	3-polyprenyl-4-hydroxybenzoate decarboxylase and related decarboxylases
Beta-galactosidase/beta-glucuronidase	ABC-type xylose transport system, permease component	Alpha-glucosidases, family 31 of glycosyl hydrolases	5-keto 4-deoxyuronate isomerase
Beta-glucanase/Beta-glucan synthetase	Antitoxin of toxin-antitoxin stability system	Alpha-mannosidase	ABC-type proline/glycine betaine transport system, ATPase component
Biotin carboxylase	Carbon dioxide concentrating mechanism/carboxysome shell protein	Amino acid permeases	ABC-type proline/glycine betaine transport system, permease component
Chromatin-associated proteins containing the HMG domain	Diadenosine tetraphosphatase and related serine/threonine protein phosphatases	ATP-dependent transcriptional regulator	ABC-type ribose transport system, auxiliary component
Flp pilus assembly protein CpaB	Flagellar basal body P-ring biosynthesis protein	Bacteriorhodopsin	Acetaldehyde dehydrogenase (acetylating)
FOG: CheY-like receiver	Gluconate kinase	Cellobiose phosphorylase	Acetyl esterase (deacetylase)
Glucitol operon activator	Mu-like prophage protein	DNA repair photolyase	Acyl CoA:acetate/3-ketoacid CoA transferase
Glutathionylspermidine synthase	Nitrate reductase alpha subunit	Ferritin-like protein	Aerobic-type carbon monoxide dehydrogenase, large subunit CoxL/CutL homologs
GTPases - Sulfate adenylate transferase subunit 1	Nitrate reductase beta subunit	High-affinity K+ transport system, ATPase chain B	Aerobic-type carbon monoxide dehydrogenase, middle subunit CoxM/CutM homologs
Holliday junction resolvasome,	Nitrate reductase delta subunit	Histone acetyltransferase	Aerobic-type carbon monoxide dehydrogenase,

endonuclease subunit			small subunit CoxS/CutS homologs
Membrane carboxypeptidase/penicillin-binding protein	Nitrate reductase gamma subunit	K+-transporting ATPase, A chain	Alkyl hydroperoxide reductase, large subunit
Na+/citrate symporter	Nucleic-acid-binding protein possibly involved in ribosomal biogenesis	K+-transporting ATPase, c chain	Alpha-galactosidases/6-phospho-beta-glucosidases, family 4 of glycosyl hydrolases
NAD-dependent protein deacetylases, SIR2 family	Plasmid stabilization system protein	Kef-type K+ transport systems, predicted NAD-binding component	Altronate dehydratase
Nucleoside-diphosphate-sugar pyrophosphorylase involved in lipopolysaccharide biosynthesis/translation initiation factor 2B, gamma/epsilon subunits (eIF-2Bgamma/eIF-2Bepsilon)	Predicted ABC-type transport system involved in lysophospholipase L1 biosynthesis, permease component	Nicotinamide mononucleotide transporter	Aminoglycoside N3'-acetyltransferase
Organic solvent tolerance protein OstA	Predicted ATPase with chaperone activity	Non-ribosomal peptide synthetase modules and related proteins	Antirestriction protein
Periplasmic protein TonB, links inner and outer membranes	Predicted endonuclease distantly related to archaeal Holliday junction resolvase and Mrr-like restriction enzymes	Peptidase E	Bacteriophytochrome (light-regulated signal transduction histidine kinase)
Phage terminase-like protein, large subunit	Predicted extracellular endo alpha-1,4 polygalactosaminidase or related polysaccharide hydrolase	Phenylacetic acid-responsive transcriptional repressor	Biopolymer transport proteins
Phosphotransferase system sorbitol-specific component IIA	Predicted membrane-associated, metal-dependent hydrolase	Phosphomannose isomerase	Cbb3-type cytochrome oxidase, subunit 1
Phosphotransferase system sorbitol-specific component IIBC	Predicted outer membrane protein	Phosphopantetheinyl transferase	Citrate lyase beta subunit
Phosphotransferase system sorbitol-specific component IIC	Predicted polymerase, most proteins contain PALM domain, HD hydrolase domain and Zn-ribbon domain	Phosphosulfolactate phosphohydrolase and related enzymes	Coenzyme F390 synthetase
Plasmid maintenance system antidote protein	Propanediol utilization protein	Polyketide synthase modules and related proteins	Cytosine/uracil/thiamine/allantoin permeases
Predicted ATPase related to phosphate starvation-inducible protein PhoH	Putative NADPH-quinone reductase (modulator of drug	Predicted ATPase/kinase involved in NAD metabolism	Demethylmenaquinone methyltransferase

	activity B)		
Predicted beta-xylosidase	Pyridoxal phosphate biosynthesis protein	Predicted glycosylase	Dihydroxyacetone kinase
Predicted deacylase	Regulator of cell morphogenesis and NO signaling	Predicted methyltransferase (contains TPR repeat)	Dipeptide/tripeptide permease
Predicted double-stranded RNA/RNA-DNA hybrid binding protein	Small integral membrane protein	Predicted P-loop ATPase fused to an acetyltransferase	DNA-directed RNA polymerase specialized sigma subunit, sigma54 homolog
Predicted nuclease of the RecB family	Spore coat assembly protein	Predicted protein-tyrosine phosphatase	D-serine dehydratase
Predicted permease, cadmium resistance protein	Transcription initiation factor IIE, alpha subunit	Predicted thioesterase involved in non-ribosomal peptide biosynthesis	Dynein, heavy chain
Predicted phage phi-C31 gp36 major capsid-like protein	Transposase and inactivated derivatives, IS30 family	Prophage antirepressor	Ferredoxin subunits of nitrite reductase and ring-hydroxylating dioxygenases
Predicted phosphatase homologous to the C-terminal domain of histone macroH2A1	Uncharacterized enzyme of heme biosynthesis	Protein related to penicillin acylase	FOG: Transposase and inactivated derivatives
Predicted phosphoesterase or phosphohydrolase	Uncharacterized membrane-bound protein conserved in bacteria	Pterin-4a-carbinolamine dehydratase	FOG: WD40-like repeat
Predicted phosphoesterases, related to the Icc protein	Uncharacterized protein conserved in archaea	Putative glucose uptake permease	Gamma-aminobutyrate permease and related permeases
Predicted phosphoribosyltransferases	Zn-ribbon protein, possibly nucleic acid-binding	Putative N-acetylmannosamine-6-phosphate epimerase	GDP-D-mannose dehydratase
Predicted protein tyrosine phosphatase		Pyrimidine reductase, riboflavin biosynthesis	Gluconolactonase
Predicted sugar isomerase		Short chain fatty acids transporter	Glucuronate isomerase
Protein involved in chromosome segregation, interacts with SMC proteins		Squalene cyclase	GTP cyclohydrolase II
Protein involved in ribonucleotide reduction		Streptogramin lyase	Histidinol phosphatase and related phosphatases
Protein-disulfide isomerase		Streptomycin 6-kinase	Homoserine trans-succinylase
Putative alpha-1,2-mannosidase		Transcriptional regulator containing an amidase domain and an AraC-type DNA-binding HTH domain	Hydantoin racemase

Putative copper export protein	Trehalose and maltose hydrolases (possible phosphorylases)	Lysine efflux permease
Putative multicopper oxidases	Type IV secretory pathway, TrbL components	Malate/L-lactate dehydrogenases
RAB proteins geranylgeranyltransferase component A (RAB escort protein)	Uncharacterized enzyme of phenylacetate metabolism	Mannose-1-phosphate guanylyltransferase
Restriction endonuclease S subunits		Metal-dependent hydrolases of the beta-lactamase superfamily II
Ribosomal protein L14E/L6E/L27E		Mn2+-dependent serine/threonine protein kinase
SH3 domain protein		Molecular chaperone, HSP90 family
Thiol:disulfide interchange protein		Na+/panthothenate symporter
Transposase		NAD(P)H-nitrite reductase
Type I restriction-modification system methyltransferase subunit		N-methylhydantoinase A/acetone carboxylase, beta subunit
Type I site-specific restriction-modification system, R (restriction) subunit and related helicases		N-methylhydantoinase B/acetone carboxylase, alpha subunit
Type IV secretory pathway, VirB4 components		Pectate lyase
Type IV secretory pathway, VirB6 components		Phosphotransferase system mannitol/fructose-specific IIA domain (Ntr-type)
Uncharacterized conserved protein related to MYG1 family		Phosphotransferase system, galactitol-specific IIC component
Uncharacterized copper-binding protein		Predicted acetyltransferase involved in intracellular survival and related acetyltransferases
Uncharacterized membrane-anchored protein		Predicted acyl-CoA transferases/carnitine dehydratase
Uncharacterized protein conserved in cyanobacteria		Predicted ATP-grasp enzyme
Uncharacterized protein with an alpha/beta hydrolase fold		Predicted epimerase, PhzC/PhzF homolog

Uncharacterized protein, homolog of Cu resistance protein CopC	Predicted metal-dependent hydrolase of the TIM-barrel fold
	Predicted nucleic acid-binding protein, contains PIN domain
	Predicted nucleotide-binding protein containing TIR -like domain
	Predicted oxidoreductase related to nitroreductase
	Predicted phosphohydrolase (DHH superfamily)
	Predicted Zn peptidase
	Prenylated rab acceptor 1 and related proteins
	Putative regulatory, ligand-binding protein related to C-terminal domains of K+ channels
	Putative stress-responsive transcriptional regulator
	Putative threonine efflux protein
	Signal transduction histidine kinase, nitrogen specific
	Sugar diacid utilization regulator
	Superfamily I DNA and RNA helicases and helicase subunits
	Tartrate dehydratase beta subunit/Fumarate hydratase class I, C-terminal domain
	Tfp pilus assembly protein FimT
	Thiamine biosynthesis protein ThiC
	Transcriptional regulators, similar to M. xanthus CarD
	Uncharacterized conserved protein (small basic protein)
	Uncharacterized MobA-related protein
	Uncharacterized protein containing SIS (Sugar ISomerase) phosphosugar

binding domain
Uncharacterized protein involved in propionate catabolism
Uncharacterized protein related to plant photosystem II stability/assembly factor
Uncharacterized protein, possibly involved in utilization of glycolate and propanediol
Uncharacterized protein/domain associated with GTPases
Uncharacterized Zn-ribbon-containing protein involved in phosphonate metabolism
Xanthine and CO dehydrogenases maturation factor, XdhC/CoxF family
Zn-dependent dipeptidase, microsomal dipeptidase homolog

Table S2. Coding DNA sequences (CDS) that are shared by *J. malaysiensis* and *J. alimentarius* genomes.

No	List of the 31 CDS shared by *J. malaysiensis* and *J. alimentarius*
1	Aspartate racemase
2	Beta- N-acetylglucosaminidase
3	cAMP-binding proteins - catabolite gene activator and regulatory subunit of cAMP-dependent protein kinases
4	Cobalamin biosynthesis protein CobT (nicotinate-mononucleotide:5, 6-dimethylbenzimidazole phosphoribosyltransferase)
5	Cobyrinic acid a,c-diamide synthase
6	Cyanate permease
7	Flavodoxins
8	Flp pilus assembly protein TadB
9	Flp pilus assembly protein TadG
10	Flp pilus assembly protein, ATPase CpaE
11	Flp pilus assembly protein, ATPase CpaF
12	Fucose permease
13	Ketosteroid isomerase homolog
14	Mg-chelatase subunit ChlD
15	Na+/melibiose symporter and related transporters
16	Predicted CDP-diglyceride synthetase/phosphatidate cytidylyltransferase
17	Predicted exporters of the RND superfamily
18	Predicted homoserine dehydrogenase
19	Predicted hydrolase of the alpha/beta superfamily
20	Predicted signal transduction protein containing EAL and modified HD-GYP domains
21	Predicted transcriptional regulator containing an HTH domain and an uncharacterized domain shared with the mammalian protein Schlafen
22	Prophage maintenance system killer protein
23	Protease II
24	Protein involved in cell division
25	Putative peptidoglycan-binding domain-containing protein
26	Putative sterol carrier protein
27	Response regulator of citrate/malate metabolism
28	Ribosomal protein L32
29	Ribosomal protein L35
30	Selenophosphate synthase
31	Uncharacterized protein related to deoxyribodipyrimidine photolyase

Table S3. Plasmid features in four genomes

**Bacteria**	**Total plasmid**	**Plasmid size (bp)**
J. malaysiensis	1	603, 070
H. halophilus	2	16, 047 and 3, 329
S. ruber	1	35, 505
H. salinarum	4	147, 625; 194, 963; 284, 332 and 40, 894

Table S4. The 224 complete pathways encoded by the *Jeotgalibacillus malaysiensis* genome.

Pathway Class	No of pathways
**Biosynthesis**	**181**
Amines and Polyamines Biosynthesis	5
Amino Acids Biosynthesis	35
Aminoacyl-tRNA Charging	2
Aromatic Compounds Biosynthesis	3
Carbohydrates Biosynthesis	8
Cell Structures Biosynthesis	5
Cofactors, Prosthetic Groups, Electron Carriers Biosynthesis	44
Fatty Acid and Lipid Biosynthesis	8
Hormones Biosynthesis	0
Metabolic Regulators Biosynthesis	2
Nucleosides and Nucleotides Biosynthesis	28
Other Biosynthesis	0
Secondary Metabolites Biosynthesis	4
Siderophore Biosynthesis	0
**Degradation/Utilization/Assimilation**	**74**
Alcohols Degradation	2
Aldehyde Degradation	1
Amines and Polyamines Degradation	1
Amino Acids Degradation	22
Aromatic Compounds Degradation	2
C1 Compounds Utilization and Assimilation	3
Carbohydrates Degradation	10
Carboxylates Degradation	6
Chlorinated Compounds Degradation	0
Cofactors, Prosthetic Groups, Electron Carriers Degradation	0
Degradation/Utilization/Assimilation - Other	1
Fatty Acid and Lipids Degradation	3
Hormones Degradation	0
Inorganic Nutrients Metabolism	4
Nucleosides and Nucleotides Degradation	8
Polymeric Compounds Degradation	2
Protein Degradation	2
Secondary Metabolites Degradation	6
Steroids Degradation	0
**Generation of Precursor Metabolites and Energy**	**20**
**Signal transduction pathways**	**0**
**Total**	**224**

Table S5. List of tRNA coded genes of *J. malaysiensis* megaplasmid (pJeoMA)

Contig accession	Feature name	Feature	Begin	End	Strand	Length	Annotation eValue	Product
8756.JMA.1.00002	JMA_t00800	tRNA	400718	400802	-	85	-1	tRNA-Ser
8756.JMA.1.00002	JMA_t00810	tRNA	401472	401542	-	71	-1	tRNA-Thr
8756.JMA.1.00002	JMA_t00820	tRNA	401820	401904	-	85	-1	tRNA-Ser
8756.JMA.1.00002	JMA_t00830	tRNA	401910	401996	-	87	-1	tRNA-Leu
8756.JMA.1.00002	JMA_t00840	tRNA	402298	402382	-	85	-1	tRNA-Leu
8756.JMA.1.00002	JMA_t00850	tRNA	402516	402586	-	71	-1	tRNA-Thr
8756.JMA.1.00002	JMA_t00860	tRNA	402830	402900	-	71	-1	tRNA-Pseudo
8756.JMA.1.00002	JMA_t00870	tRNA	403435	403519	-	85	-1	tRNA-Ser
8756.JMA.1.00002	JMA_t00880	tRNA	403933	404005	-	73	-1	tRNA-Phe
8756.JMA.1.00002	JMA_t00890	tRNA	404162	404236	-	75	-1	tRNA-Undet
8756.JMA.1.00002	JMA_t00900	tRNA	404329	404404	-	76	-1	tRNA-Asn
8756.JMA.1.00002	JMA_t00910	tRNA	404411	404485	-	75	-1	tRNA-Asp
8756.JMA.1.00002	JMA_t00920	tRNA	404488	404562	-	75	-1	tRNA-Asn
8756.JMA.1.00002	JMA_t00930	tRNA	404568	404642	-	75	-1	tRNA-Ile
8756.JMA.1.00002	JMA_t00940	tRNA	404644	404718	-	75	-1	tRNA-Asn
8756.JMA.1.00002	JMA_t00950	tRNA	404724	404800	-	77	-1	tRNA-Gln
8756.JMA.1.00002	JMA_t00960	tRNA	405363	405433	-	71	-1	tRNA-Gly
8756.JMA.1.00002	JMA_t00970	tRNA	405622	405697	-	76	-1	tRNA-Pseudo
8756.JMA.1.00002	JMA_t00980	tRNA	405767	405854	-	88	-1	tRNA-Cys

8756.JMA.1.00002	JMA_t00990	tRNA	405859	405932	-	74	-1	tRNA-Gly
8756.JMA.1.00002	JMA_t01000	tRNA	406276	406347	-	72	-1	tRNA-Asp
8756.JMA.1.00002	JMA_t01010	tRNA	406434	406507	-	74	-1	tRNA-Phe
8756.JMA.1.00002	JMA_t01020	tRNA	406516	406591	-	76	-1	tRNA-Thr
8756.JMA.1.00002	JMA_t01030	tRNA	406596	406669	-	74	-1	tRNA-Val
8756.JMA.1.00002	JMA_t01040	tRNA	406675	406746	-	72	-1	tRNA-Arg
8756.JMA.1.00002	JMA_t01050	tRNA	406748	406821	-	74	-1	tRNA-Ala
8756.JMA.1.00002	JMA_t01060	tRNA	406824	406900	-	77	-1	tRNA-Pro
8756.JMA.1.00002	JMA_t01070	tRNA	406906	406990	-	85	-1	tRNA-Tyr
8756.JMA.1.00002	JMA_t01080	tRNA	407254	407325	-	72	-1	tRNA-Glu
8756.JMA.1.00002	JMA_t01090	tRNA	407337	407410	-	74	-1	tRNA-Trp
8756.JMA.1.00002	JMA_t01100	tRNA	407417	407488	-	72	-1	tRNA-Ala
8756.JMA.1.00002	JMA_t01110	tRNA	407490	407566	-	77	-1	tRNA-Pro
8756.JMA.1.00002	JMA_t01120	tRNA	407571	407645	-	75	-1	tRNA-Glu
8756.JMA.1.00002	JMA_t01130	tRNA	407647	407723	-	77	-1	tRNA-Pro
8756.JMA.1.00002	JMA_t01140	tRNA	407729	407803	-	75	-1	tRNA-Ile
8756.JMA.1.00002	JMA_t01150	tRNA	407806	407879	-	74	-1	tRNA-His
8756.JMA.1.00002	JMA_t01160	tRNA	408088	408160	-	73	-1	tRNA-Lys
8756.JMA.1.00002	JMA_t01170	tRNA	411266	411341	-	76	-1	tRNA-Arg
8756.JMA.1.00002	JMA_t01180	tRNA	411351	411431	-	81	-1	tRNA-Leu
8756.JMA.1.00002	JMA_t01190	tRNA	411611	411681	-	71	-1	tRNA-Thr

Table S6. Supplimentary table for Fig. 4

Box No	Enzymes/Gene Name	Gene locus ID
**TCA**		
1	Aconitate hydratase	JMA_18040
2	Aconitate hydratase	JMA_18040
3	Isocitrate dehydrogenase	JMA_24230
4	2-oxoglutarate dehydrogenase E2 component (dihydrolipoamide succinyltransferase)	JMA_18720
5	Succinyl-CoA synthetase alpha subunit	JMA_16660
6	Succinate dehydrogenase / fumarate reductase	JMA_23770
		JMA_23760
7	Fumarate hydratase, class II	JMA_12470
		JMA_13220
8	Malate dehydrogenase (quinone)	JMA_24220
9	Malate dehydrogenase	JMA_24220
**Glycolysis**		
10	Phosphoglucomutase	JMA_08750
11	Glucose-6-phosphate isomerase	JMA_25610
12	Glucose-6-phosphate isomerase	JMA_25610
13	6-phosphofructokinase 1	JMA_24290
14	Fructose-bisphosphate aldolase, class I	JMA_35110
15	Glyceraldehyde 3-phosphate dehydrogenase	JMA_26950
16	Phosphoglycerate kinase	JMA_26940
17	2,3-bisphosphoglycerate-independent phosphoglycerate mutase	JMA_26910
18	Enolase	JMA_26910
19	Pyruvate kinase	JMA_24280
**PP Pathways**		
20	Glucose-6-phosphate 1-dehydrogenase	JMA_20600
21	6-phosphogluconolactonase	JMA_07020
22	Glucose-6-phosphate isomerase	JMA_25610
23	6-phosphogluconate dehydrogenase	JMA_20620
24	3-hexulose-6-phosphate synthase	JMA_08740
25	6-phospho-3-hexuloisomerase	JMA_29160
26	Glucose-6-phosphate isomerase	JMA_25610
27	Fructose-1,6-bisphosphatase II	JMA_30420
28	6-phosphofructokinase 1	JMA_24290
29	Fructose-bisphosphate aldolase, class I	JMA_35110
30	Transketolase	JMA_17860

31	Ribulose-phosphate 3-epimerase	JMA_16370
32	Transaldolase	JMA_30440
33	Ribose 5-phosphate isomerase A	JMA_30200
34	Ribose-phosphate pyrophosphokinase	JMA_00670
35	Transketolase	JMA_17860
**ABC transporter Alkanosulfonate**		
36	Sulfonate transport system substrate-binding protein	JMA_07420
37	Sulfonate transport system permease protein	JMA_07250
38	Sulfonate transport system ATP-binding protein	JMA_07260
**Molybdate**		
39	Molybdate transport system substrate-binding protein	JMA_04750
40	Molybdate transport system permease protein	JMA_04760
**Iron (III)**		
41	Iron(III) transport system substrate-binding protein	JMA_31010
42	Iron(III) transport system permease protein	JMA_31030
43	Iron(III) transport system ATP-binding protein	JMA_31040
**Osmoprotectant**		
44	Osmoprotectant transport system substrate-binding protein	JMA_18130
45	Osmoprotectant transport system permease protein	JMA_18120
46	Osmoprotectant transport system ATP-binding protein	JMA_18110
**Iron complex**		
47	Iron complex transport system substrate-binding protein	JMA_05610
48	Iron complex transport system permease protein	JMA_05580
49	Iron complex transport system ATP-binding protein	JMA_05600
**Zinc**		
50	Zinc transport system substrate-binding protein	JMA_25460
51	Zinc transport system permease protein	JMA_21580
52	Zinc transport system ATP-binding protein	JMA_21590
**Biotin**		
53	Biotin transport system substrate-specific component	JMA_27700
54	Energy-coupling factor transport system permease protein	JMA_01660
55	Energy-coupling factor transport system ATP-binding protein	JMA_01640
56	Energy-coupling factor transport system ATP-binding protein	JMA_01650
**Phosphate**		
57	Phosphate transport system substrate-binding protein PstS	JMA_21470
58	Phosphate transport system permease protein PstC	JMA_21460

59	Phosphate transport system permease protein PstA	JMA_21450
60	Phosphate transport system ATP-binding protein PstB	JMA_21440
**Cystine**		
61	Cystine transport system substrate-binding protein FliY	JMA_07080
62	Cystine transport system permease protein YecS	JMA_07070
63	Cystine transport system ATP-binding protein YecC	JMA_07640
**Methionine**		
64	D-methionine transport system substrate-binding protein MetQ	JMA_26330
65	D-methionine transport system permease protein MetI	JMA_26340
66	D-methionine transport system ATP-binding protein MetN	JMA_26350
**Oligopeptide**		
67	Oligopeptide transport system substrate-binding protein OppA	JMA_31260
68	Oligopeptide transport system permease protein OppB	JMA_31240
69	Oligopeptide transport system permease protein OppC	JMA_31250
70	Oligopeptide transport system ATP-binding protein OppD	JMA_31230
71	Oligopeptide transport system ATP-binding protein OppF	JMA_31220
**Proline synthesis**		
72	Gamma-glutamyl kinase	JMA_18220
73	Gamma_glutamyl phosphate reductase	JMA_18230
74	Pyrroline-5-carboxylate reductase	JMA_18210
**Trehalose synthesis**		
75	Phosphoglucomutase	JMA_12960
76	Glucose-1-phosphate adenyltransferase	JMA_11400
77	Glycogen synthase	JMA_11380
78	Maltooligosyl-trehalose synthase	JMA_13850
79	Alpha amylase	JMA_33490
**Sporulation**		
80	Sporulation sensor kinase B	JMA_14220
81	Kinase-associated protein B	JMA_25770
82	Stage 0 sporulation protein F	JMA_30470
83	Stage 0 sporulation protein B	JMA_23290
84	Stage 0 sporulation protein A	JMA_20860

Table S7 RPKM, TMM, and RLE values
Note:
1. Only genes that are up- or down-regulated are shown.
2. RPKM 2, RPKM 10, and RPKM 20 refer to the values respectively for 2% NaCl, 10% NaCl, and 20% NaCl.

Gene name	Annotation	Genome	RPKM 2	RPKM 10	RPKM 20	TMM 2	TMM 10	TMM 20	RLE 2	RLE 10	RLE 20
JMA_RS03200	Molybdopterin molybdenumtransferase	JMA_2684(	16100.67	60.5	574.335	234294.435	713	8176.79	206294.565	620.535	7158.78
JMA_RS04515	metallophosphoesterase	JMA_0897(	916.675	4.47	48.905	4402.38	17.375	237.005	3871.33	15.11	208.545
JMA_RS17735	Binds together with S18 to 16S ribosomal RNA {ECO:0000255|HAMAP-Rule:MF_00360}	JMA_3628(	1675.085	10.11	60.165	32965.855	161.4	1159.995	29014.735	140.52	1016.125
JMA_RS04540	ABC transporter substrate-binding protein|Maltose-binding periplasmic protein|Involved in the high-affinity maltose membrane transport system MalEFGK Initial receptor for the active transport of and chemotaxis toward maltooligosaccharides (By similarity) {ECO:0000250}	JMA_0902(	1813.335	11.58	76.4	19633.14	101.985	821.25	17281.025	88.815	721.135
JMA_RS04520	phosphoglycerate mutase	JMA_0898(	528.975	3.385	31.875	6213.86	32.07	380.2	5467.075	27.895	335.44
JMA_RS04510	2'-5' RNA ligase	JMA_0896(	581.985	4.04	27.02	1707	9.635	81.575	1501.91	8.385	71.895
JMA_RS04525	Catalyzes the ATP-dependent amidation of the two carboxylate groups at positions a and c of cobyrinate, using either L-glutamine or ammonia as the nitrogen source {ECO:0000255|HAMAP-Rule:MF_00027}|2 ATP + cobyrinate + 2 L-glutamine + 2 H(2)O = 2 ADP + 2 phosphate + cobyrinate a,c-diamide + 2 L-glutamate {ECO:0000255|HAMAP-Rule:MF_00027}|cobyrinic acid a,c-diamide synthase|Cobyrinate a,c-diamide synthase {ECO:0000255|HAMAP-Rule:MF_00027}	JMA_0899(	403.635	2.89	38.175	1070.515	6.205	103.685	942.44	5.385	91.26
JMA_RS17400	SAM-dependent methyltransferase	JMA_3564(	985.155	9.085	40.53	15359.19	114.58	626.28	13518.905	99.705	549.915
JMA_RS13030	Derived by automated computational analysis using gene prediction method: Protein Homology.|hypothetical protein	JMA_2644(	836.585	8.215	94.545	24500.455	195.22	2832.82	21564.01	169.985	2505.595
JMA_RS14995	Ddeacetylase|Uncharacterized deacetylase YojG	JMA_3059(	640.71	6.325	37.95	8593.93	68.65	512.58	7561.81	59.74	451.54
JMA_RS12910	hypothetical protein	JMA_2691(	1005.445	10.615	159.84	18075.69	154.3	2958.66	15905.94	134.27	2619.215
JMA_RS14985	hypothetical protein	JMA_3057(	179.55	2.265	11.58	2308.435	23.54	151.885	2031.255	20.48	134.155
JMA_RS15675	hypothetical protein	JMA_2873(	1140.535	17.68	278.465	17379.15	218.97	4386.095	15293.42	190.695	3885.055
JMA_RS08630	glycine/betaine ABC transporter permease|Glycine betaine transporter OpuD|High-affinity uptake of glycine betaine Does not mediate either carnitine or choline uptake {ECO:0000269|PubMed:8752321}	JMA_1740(	3317.98	53.205	254.77	24603.525	321.235	1940.255	21652.765	279.79	1713.975
JMA_RS14990	hypothetical protein|Uncharacterized protein YojF	JMA_3058(	288.35	4.97	17.895	2620.955	36.61	165.79	2305.455	31.865	146.33
JMA_RS13025	hypothetical protein	JMA_2643(	562.1	9.9	62.91	7538.775	107.815	863.22	6633.86	93.875	762.885
JMA_RS10615	terminase	JMA_2168(	517.72	9.755	86.445	6696.815	101.915	1158.64	5893.02	88.665	1026.505
JMA_RS06755	adapter protein MecA|Adapter protein MecA 1	JMA_1357(	768.915	17.095	88.6	14121.495	254.125	1657.445	12431.47	221.165	1463.9
JMA_RS15680	hypothetical protein	JMA_3198(	373.985	10.14	56.295	5743.21	126.065	890.11	5053.945	109.725	787.805
JMA_RS17415	tRNA-dihydrouridine synthase|Probable tRNA-dihydrouridine synthase 2|Catalyzes the synthesis of dihydrouridine, a modified base found in the D-loop of most tRNAs {ECO:0000250}	JMA_3199(	202.975	7.455	14	2527.39	75.19	173.605	2224.96	65.435	152.56
JMA_RS10040	3-dehydroquinate dehydratase	JMA_2047(	230.585	8.54	40.585	1653.03	49.465	300.71	1455.79	43.02	265.87
JMA_RS12645	Uncharacterized metallophosphoesterase YunD	JMA_2565(	1412.015	63.685	115.825	19153.165	700.545	1596.52	16859.94	609.835	1408.905
JMA_RS12130	hypothetical protein	JMA_2485(	1003.02	47.21	276.065	20617.725	783.505	5859.04	18144.945	681.685	5190.555
JMA_RS11650	universal stress protein UspA|Putative universal stress protein SSP1056	JMA_2385(	1248.365	66.73	66.635	10046.94	437.41	540.66	8843.605	380.98	475.995
JMA_RS14970	Uracil-DNA glycosylase	JMA_3054(	249.115	14.155	25.385	10204.39	469.185	1059.31	8980.095	408.365	936.31
JMA_RS17170	hypothetical protein	JMA_3518(	662.025	41.26	1330.14	8767.485	442.645	18444.545	7719.275	385.25	16373.055
JMA_RS15525	hypothetical protein	JMA_3168(	5331.11	350.105	9266.14	3847.4	207.26	8005.425	3387.515	180.325	7123.51
JMA_RS08975	Anthranilate phosphoribosyltransferase	JMA_1812(	1390.31	93.545	1421.045	8870.83	485.045	9619.05	7808.755	422.265	8542.23
JMA_RS14580	ABC transporter permease	JMA_2970(	2613.11	183.58	7761.925	10039.505	573.14	32062.385	8836.175	498.775	28471.895
JMA_RS12650	transmembrane protein	JMA_2566(	2334.295	170.81	197.215	42685.66	2536.1	3660.495	37576.925	2208.21	3230.655
JMA_RS08970	Indole-3-glycerol phosphate synthase	JMA_1811(	1742.995	130.18	1934.165	18522.535	1123.515	21536.525	16305.355	978.185	19111.84
JMA_RS11845	Acetyl-coenzyme A synthetase	JMA_0238(	2051.86	162.545	202.87	25798.26	1661.58	2602.195	22708.185	1446.985	2297.985
JMA_RS09220	Ribonuclease H	JMA_1873(	1036.705	92.215	110.19	15131.1	1093.45	1642.65	13320.135	952.12	1451.235
JMA_RS12380	hypothetical protein	JMA_2536(	1077.49	100.305	141.895	20344.06	1534.445	2735.79	17909.735	1335.635	2417.67
JMA_RS03185	Molybdopterin-synthase adenylyltransferase	JMA_0629(	463.235	46.025	357.455	10309.055	830.63	8260.34	9075.435	723.12	7326.68
JMA_RS08270	Uncharacterized protein YlxP	JMA_1667(	2579.335	258.94	110.9	25157.635	2053.735	1070.365	22146.5	1788.395	939.11
JMA_RS11615	oligoribonuclease	JMA_2378(	1238.405	124.375	267.46	7263.93	593.415	1644.9	6393.605	516.69	1457.4
JMA_RS11605	Oxaloacetate = pyruvate + CO(2)|malate dehydrogenase|Probable NAD-dependent malic enzyme 4	JMA_2376(	4034.61	416.505	1335.91	32462.39	2723.505	11253.35	28579.115	2371.2	9977.31
JMA_RS01790	hypothetical protein	JMA_0525(	159.31	16.585	16.395	3956.83	333.19	411.665	3483.005	289.975	363.14
JMA_RS10170	Lipoteichoic acid synthase 2	JMA_2075(	119.14	13.07	36.5	2302.45	204.39	731.615	2026.255	177.88	648.715
JMA_RS11610	DNA polymerase III subunit alpha	JMA_2377(	2542.96	282.695	784.1	53675.955	4843.185	17094.485	47245.25	4216.985	15145.19
JMA_RS17135	Glycerol uptake operon antiterminator regulatory protein	JMA_3511(	7222.97	830.61	12871.645	69588.14	6469.265	129865.165	61259.905	5628.065	115203.51
JMA_RS08265	Translation initiation factor IF-2	JMA_1666(	1297.125	163.06	57.93	17081.465	1747.64	752.17	15035.27	1522.2	659.64
JMA_RS13035	hypothetical protein	JMA_3154(	506.85	67.555	60.5	5044.465	545.35	614.53	4440.485	474.705	542.515
JMA_RS02695	short-chain dehydrogenase|Dehydrogenase/reductase SDR family member 13|Putative oxidoreductase	JMA_0653(	44.87	6.025	11.115	881.705	95.93	223.685	775.72	83.51	197.82
JMA_RS08980	Aminodeoxychorismate synthase component 2	JMA_1813(	1788.705	246.94	3081.705	17238.24	1929.57	31206.8	15172.005	1679.55	27702.76
JMA_RS05650	hypothetical protein	JMA_1140(	834.76	116.21	140.68	10961.3	1233.11	1892.555	9647.14	1072.875	1672.905
JMA_RS06575	hypothetical protein|Uncharacterized protein YxaH|Involved in transport	JMA_1322(	1064.065	151.52	264.26	17220.99	1988.82	4410.42	15158.94	1731.38	3904.77
JMA_RS05645	hypothetical protein|Probable transcriptional regulatory protein GbCGDNIH1_1097	JMA_1141(	956.89	154.47	185.88	10893.5	1419.615	2175.88	9587.52	1234.9	1924.265
JMA_RS04635	histidine kinase|Sensor protein BceS	JMA_0922(	302.68	52.395	92.085	5380.05	753.535	1695.87	4735.165	655.795	1503.15
JMA_RS14845	Hypothetical protein|Uncharacterized beta-barrel protein YwiB	JMA_3026(	49.17	8.52	59.14	555.71	77.935	700.815	489.18	67.82	622.095
JMA_RS05655	Peptidase S8|Alkaline protease	JMA_3661(	766.675	133.56	167.02	17341.27	2442.915	3869.66	15262.09	2125.965	3422.8
JMA_RS11835	Tyrosine--tRNA ligase	JMA_2424(	1536.44	289.67	149.255	15837.65	2422.78	1568.78	13943.115	2109.015	1384.72
JMA_RS17900	hypothetical protein	JMA_1822(	1130.465	216.1	310.445	11922.8	1843.295	3375.91	10496.095	1603.765	2986.48
JMA_RS05640	short-chain dehydrogenase|Uncharacterized oxidoreductase YgfF	JMA_1138(	354.655	70.765	49.285	5980.675	965.6	847.635	5263.445	840.36	748.825

JMA_RS15855	D-aminopeptidase	JMA_3236(	1513.06	302.18	320.71	19500.245	3156.115	4257.6	17167.185	2747.05	3767.14
JMA_RS16700	Putative fluoride ion transporter CrcB 2	JMA_3418(	510.685	103.925	102.89	8933.76	1476.305	1849.86	7864.125	1285.455	1636.8
JMA_RS11840	peptidoglycan glycosyltransferase	JMA_2423(	3035.85	695.4	234.555	44307.61	8240.835	3429.27	39005.825	7175.1	3017.54
JMA_RS17140	hypothetical protein	JMA_3512(	474.405	123.98	928.73	8598.275	1821.945	17537.215	7570.32	1586.01	15560.82
JMA_RS14920	octanoyltransferase	JMA_3042(	1207.915	354.985	103.34	12740.575	3040.055	1094.285	11215.46	2646.705	962.98
JMA_RS10295	endonuclease IV	JMA_2103(	584.27	183.165	45.225	2546.805	650.685	200.655	2241.255	566.7	176.73
JMA_RS05635	hypothetical protein	JMA_1137(	280.21	100.48	44.03	8239.52	2381.145	1305.115	7252.08	2071.22	1150.665
JMA_RS07555	hypothetical protein|Uncharacterized N-acetyltransferase ABC2369	JMA_1522(	3240.51	1168.77	1280.91	34830.045	10170.37	14257.93	30656.05	8850.725	12625.145
JMA_RS12655	hypothetical protein|UPF0759 protein YunF	JMA_2567(	89.23	33.06	15.605	1355.96	405.445	243.415	1193.305	352.7	215.29
JMA_RS08915	voltage-gated sodium channel	JMA_1779(	1349.38	590.93	966.255	24568.095	8732.89	18257.51	21627.65	7603.44	16187.745
JMA_RS12700	Methionine import ATP-binding protein MetN 2	JMA_2646(	341.31	159.195	65.21	2435.485	920.93	475.465	2143.675	801.465	419.56
JMA_RS16705	glyoxalase	JMA_3419(	197.015	94.29	101.73	11502.555	4465.735	6128.305	10123.96	3888.355	5432.78
JMA_RS03770	Putative carboxypeptidase YodJ	JMA_0753(	681.34	331.24	1461.67	12623.93	4957.905	28180.935	11111.33	4313.765	25001.49
JMA_RS17335	hypothetical protein	JMA_3550(	36.605	20.695	93.39	332.12	151.78	892.745	292.495	132.025	792.64
JMA_RS15460	gas vesicle protein GvpG	JMA_3154(	510.025	289.39	992.56	4346.845	1997.325	8815.045	3826.675	1737.995	7810.055
JMA_RS14295	hypothetical protein	JMA_2952(	20.31	11.56	18.88	301.665	138.56	291.535	265.305	120.555	258.555
JMA_RS14290	hypothetical protein	JMA_2912(	23.535	14.095	17.3	38.61	18.87	30.85	33.965	16.43	27.32
JMA_RS03210	molybdenum cofactor biosynthesis protein MoaE|Molybdopterin synthase catalytic subunit	JMA_0636(	2805.955	1792.07	1361.58	9990.01	5197.755	5130.935	8797.02	4524.295	4543.11
JMA_RS03215	molybdenum cofactor biosynthesis protein MoaD	JMA_0789(	178.315	115.905	200.06	1789.31	939.98	2098.63	1574.795	817.78	1861.385
JMA_RS10000	hypothetical protein|Uncharacterized protein YqhY	JMA_2038(	1206.355	830.92	6933.325	16315.995	9107.08	98036.335	14362.24	7926.945	87008.375
JMA_RS07525	hypothetical protein|Uncharacterized protein YlbL	JMA_1516(	736.155	550.295	445.12	4815.735	2921.12	3066.845	4238.22	2542.575	2720.5
JMA_RS00165	Nucleoid-associated protein Tfu_0045	JMA_0031(	117.795	88.965	40.53	2060.79	1262.71	727.395	1813.99	1099.33	643.38
JMA_RS13780	hypothetical protein	JMA_2806(	7.685	6.08	9.145	72.535	46.085	89.3	63.835	40.055	79.03
JMA_RS17795	Derived by automated computational analysis using gene prediction method: Protein Homology.|tRNA modification GTPase|tRNA modification GTPase MnmE {ECO:0000255|HAMAP-Rule:MF_00379}|Exhibits a very high intrinsic GTPase hydrolysis rate Involved in the addition of a carboxymethylaminomethyl (cmnm) group at the wobble position (U34) of certain tRNAs, forming tRNA- cmnm(5)s(2)U34 {ECO:0000255|HAMAP-Rule:MF_00379}	JMA_1139(	117.745	93.755	67.975	751.055	484.67	453.11	661.245	421.71	401.31
JMA_RS09410	Derived by automated computational analysis using gene prediction method: Protein Homology.|Zn-dependent protease|Putative membrane protease YugP	JMA_1913(	512.32	412.475	1697.14	2516.76	1642.97	8888.195	2215.32	1429.48	7891.39
JMA_RS12640	Member of the two-component regulatory system RpfG/RpfC, which is required for full virulence and for formation and dispersal of biofilms Involved in sensing and responding to the diffusible signaling factor (DSF), which is essential for cell- cell signaling Activated RpfG degrades cyclic di-GMP, an unusual nucleotide second messenger, leading to the activation of Clp, a global transcriptional regulator that regulates a large set of genes in DSF pathway May also directly control genes involved in biofilm dispersal {ECO:0000269|PubMed:11123673, ECO:0000269|PubMed:12960398, ECO:0000269|PubMed:15158198, ECO:0000269|PubMed:16611728, ECO:0000269|PubMed:17378922, ECO:0000269|PubMed:20231439}|Cyclic di-3',5'-guanylate + H(2)O = 5'- phosphoguanylyl(3'->5')guanosine {ECO:0000269|PubMed:16611728}|histidine kinase|Cyclic di-GMP phosphodiesterase response	JMA_2564(	1706.15	1382.445	546.435	24362.785	15988.83	7986.96	21445.205	13916.045	7059.345
JMA_RS13975	Derived by automated computational analysis using gene prediction method: Protein Homology.|hypothetical protein|Ig-like virion protein {ECO:0000312|EMBL:BAH151781}	JMA_2844(	215.995	181.13	68.895	4345.89	2955.575	1418.32	3824.425	2573.19	1254.1
JMA_RS14815	Derived by automated computational analysis using gene prediction method: Protein Homology.|SAM-dependent methyltransferase|Uncharacterized methyltransferase OB1106 {ECO:0000255|HAMAP-Rule:MF_02100}|Could be a S-adenosyl-L-methionine-dependent methyltransferase {ECO:0000255|HAMAP-Rule:MF_02100}	JMA_3020(	332.12	303.055	980.625	1209.455	898.695	3815.77	1064.76	782.3	3384.1
JMA_RS12365	L-glutamate 5-semialdehyde + NAD(+) + H(2)O = L-glutamate + NADH|1-pyrroline-5-carboxylate dehydrogenase|1-pyrroline-5-carboxylate dehydrogenase 1	JMA_2533(	197.215	184.255	432.625	3474.19	2628.905	7928.78	3057.76	2288.235	7033.5
JMA_RS06420	Derived by automated computational analysis using gene prediction method: Protein Homology.|hypothetical protein|Protein EcsB|Presumed to form part of an ABC-transporter, it may form a transport channel {ECO:0000269|PubMed:15175311}	JMA_1287(	55.235	53.005	30.82	1078.085	837.73	613.37	948.995	729.14	541.915

JMA_RS00455	step biosynthesis of 4-amino-4-deoxychorismate (ADC), a precursor of p-aminobenzoate (PABA) and tetrahydrofolate In the first step, a glutamine amidotransferase (PabA) generates ammonia as a substrate that, along with chorismate, is used in the second step, catalyzed by aminodeoxychorismate synthase (PabB) to produce ADC PabA converts glutamine into glutamate only in the presence of stoichiometric amounts of PabB Also involved in the biosynthesis of anthranilate {ECO:0000269|PubMed:2123867}|Chorismate + L-glutamine = 4-amino-4- deoxychorismate + L-glutamate|anthranilate synthase component II|Aminodeoxychorismate/anthranilate synthase component 2	JMA_3567(	143.625	145.19	64.615	1412.86	1160.725	653.765	1243.39	1010.735	578.04
JMA_RS04460	Derived by automated computational analysis using gene prediction method: Protein Homology.|choline transporter|Glycine betaine transporter OpuD|High-affinity uptake of glycine betaine Does not mediate either carnitine or choline uptake {ECO:0000269|PubMed:8752321}	JMA_1912(	78.68	82.995	27.845	445.92	382.23	164.22	392.475	332.745	145.295
JMA_RS09425	electron transport protein; Derived by automated computational analysis using gene prediction method: Protein Homology.|cytochrome b6|Menaquinol-cytochrome c reductase cytochrome b subunit|Component of the menaquinol-cytochrome c reductase complex	JMA_1906(	325.095	351.31	710.86	2823.555	2472.7	6460.8	2484.965	2152.075	5729.445
JMA_RS06895	Phosphorylates the sporulation-regulatory protein spo0A|ATP + protein L-histidine = ADP + protein N-phospho-L-histidine|hypothetical protein|Sporulation kinase C	JMA_1385(	74.59	90.735	22.21	1562.25	1537.61	467.89	1375.325	1338.145	412.215
JMA_RS14030	Catalyzes the condensation of ribulose 5-phosphate with formaldehyde to form 3-hexulose 6-phosphate {ECO:0000269|PubMed:10418139}|D-arabino-hex-3-ulose 6-phosphate = D-ribulose 5-phosphate + formaldehyde|Fe-S cluster assembly protein HesB|3-hexulose-6-phosphate synthase	JMA_2856(	68.195	92	49.64	1628.7	1775.865	1213.01	1433.745	1545.29	1072.67
JMA_RS09100	Cleavage of adenosine and its derivatives|Purine deoxynucleoside + phosphate = purine + 2'-deoxy-alpha-D-ribose 1-phosphate|purine nucleoside phosphorylase|Purine nucleoside phosphorylase DeoD-type	JMA_1840(	338.87	461.015	1176.57	1610.01	1779.225	5953.615	1417.575	1548.36	5283.835
JMA_RS14285	Derived by automated computational analysis using gene prediction method: Protein Homology.|hypothetical protein|Peptidoglycan endopeptidase LytF|Cell wall hydrolase that cleaves gamma-D-glutamate-meso- diaminopimelate bonds in peptidoglycan LytF is necessary and sufficient for vegetative daughter cell separation, and also seems to play a role in cell autolysis {ECO:0000269|PubMed:10206711, ECO:0000269|PubMed:10322020, ECO:0000269|PubMed:19542270}	JMA_2911(	16.98	23.135	20.07	114.43	126.21	142.175	100.71	109.81	126.085
JMA_RS17965	Derived by automated computational analysis using gene prediction method: GeneMarkS+.|hypothetical protein	JMA_3672(	178.175	281.62	536.02	2063.045	2641.17	6476.585	1816.18	2298.6	5743.99
JMA_RS03930	Derived by automated computational analysis using gene prediction method: Protein Homology.|antitoxin EndoAI|Antitoxin EndoAI|Antitoxin component of a toxin-antitoxin (TA) module Antitoxin that directly inhibits activity of EndoA in vitro Upon expression in Ecoli counteracts inhibitory effect of mRNA interferase EndoA {ECO:0000269|PubMed:15882409, ECO:0000269|PubMed:21763692}	JMA_1142(	97.005	159.935	60.44	557.83	748.76	363.415	490.8	652.13	321.85
JMA_RS21910	Flagellin and realed hook associated protein	JMA_4441(	0.635	1.06	3.44	5.68	7.69	31.935	5	6.7	28.29
JMA_RS18035	Derived by automated computational analysis using gene prediction method: Protein Homology.|hypothetical protein	JMA_3686(	194.255	339.915	179.945	1871.62	2650.95	1788.24	1647.54	2306.735	1581.79
JMA_RS14110	May be involved in synthesis of N-acetyltrideoxygalactose, a component of exopolysaccharide EPS I which functions as a virulence factor|UDP-N-acetyl-alpha-D-glucosamine = UDP-N- acetyl-alpha-D-mannosamine|UDP-N-acetylglucosamine 2-epimerase|Probable UDP-N-acetylglucosamine 2-epimerase	JMA_2845(	14.94	26.96	32.645	298.825	435.89	677.445	262.965	379.315	600.74
JMA_RS05105	Derived by automated computational analysis using gene prediction method: Protein Homology.|membrane protein|Uncharacterized membrane protein YdjJ	JMA_1026(	500.08	924.7	1465.385	5033.48	7550.88	15392.92	4432.755	6572.31	13646.23
JMA_RS12630	Derived by automated computational analysis using gene prediction method: Protein Homology.|sodium:proton antiporter|Uncharacterized protein HI_1586	JMA_2562(	528.495	987.13	1890.255	6977.72	10585.39	26018.175	6143.7	9216.355	23076.005
JMA_RS09255	Catalyzes the reduction of 2,5-diketo-3-deoxygluconate (DKII or 4,6-dihydroxy-2,5-dioxohexanoate) into 2-keto-3- deoxygluconate (KDG or 2-dehydro-3-deoxygluconate) with a concomitant oxidation of NADH {ECO:0000269|PubMed:1766386}|2-dehydro-3-deoxy-D-gluconate + NAD(+) = (4S)- 4,6-dihydroxy-2,5-dioxohexanoate + NADH {ECO:0000269|PubMed:1766386}|2-deoxy-D-gluconate 3-dehydrogenase|2-dehydro-3-deoxy-D-gluconate 5-dehydrogenase	JMA_1880(	40.685	84.42	30.99	328.965	551.6	260.02	289.63	479.785	230.165

JMA_RS06645	Derived by automated computational analysis using gene prediction method: Protein Homology.|LacI family transcriptional regulator|HTH-type transcriptional regulator MalR|Transcriptional repressor of the maltosaccharide utilization operons malxCD and malMP {ECO:0000250}	JMA_1717(	237.875	538.41	203.22	2396.11	4387.91	2106.19	2109.045	3818.045	1862.38
JMA_RS12625	Derived by automated computational analysis using gene prediction method: Protein Homology.|transporter|Sodium- and chloride-dependent betaine transporter|Transports betaine and GABA May have a role in regulation of GABAergic transmission in the brain through the reuptake of GABA into presynaptic terminals, as well as in osmotic regulation	JMA_2561(	481.88	1121.455	240.05	7547.445	14280.285	3852.895	6645.6	12435.625	3406.375
JMA_RS15830	Derived by automated computational analysis using gene prediction method: Protein Homology.|peptidase S66|Probable murein peptide carboxypeptidase|May be involved in the degradation of peptidoglycan by catalyzing the cleavage of the terminal D-alanine residue from cytoplasmic murein peptides {ECO:0000250}	JMA_3230(	60.51	151.625	79.355	926.35	1874.435	1247.175	815.545	1630.675	1103.07
JMA_RS08520	Derived by automated computational analysis using gene prediction method: Protein Homology.|membrane protein|UPF0126 membrane protein YvgT	JMA_1821(	190.35	527.695	205.205	3895.97	8730.38	4309.62	3429.945	7596.635	3812.635
JMA_RS10300	ATP-binding RNA helicase involved in the biogenesis of 60S ribosomal subunits and is required for the normal formation of 25S and 58S rRNAs {ECO:0000250}|ATP + H(2)O = ADP + phosphate|DEAD/DEAH box helicase|ATP-dependent RNA helicase dbp7	JMA_2520(	4.91	14.98	10.375	23.11	57.175	52.475	20.355	49.73	46.625
JMA_RS10075	Derived by automated computational analysis using gene prediction method: Protein Homology.|hypothetical protein|Uncharacterized protein YqhL	JMA_2056(	33.17	103.47	33.57	284.005	718.845	296.435	250.03	625.71	262.135
JMA_RS21915	Flagellin and realed hook associated protein	JMA_4442(	0.445	1.43	3.3	4.01	10.415	31.255	3.525	9.065	27.75
JMA_RS15225	Derived by automated computational analysis using gene prediction method: GeneMarkS+.|hypothetical protein	JMA_2299(	708.715	2302.13	1687.51	7557.76	19872.36	18614.32	6654.185	17292.11	16477.145
JMA_RS05915	Possesses glyoxalase I activity Catalyzes the conversion of hemimercaptal, formed from methylglyoxal and glutathione, to S-lactoylglutathione May be involved in oxidative stress response {ECO:0000269|PubMed:23651081}|(R)-S-lactoylglutathione = glutathione + methylglyoxal|cysteine protease|Protein DJ-1 homolog D	JMA_1192(	206.64	705.58	476	1342.805	3716.29	3246.1	1182.17	3233.72	2877.345
JMA_RS10095	Derived by automated computational analysis using gene prediction method: GeneMarkS+.|hypothetical protein	JMA_2060(		702.39	143.725	3456.94	10004.89	2567.765	3043.175	8709.18	2266.945
JMA_RS15365	Derived by automated computational analysis using gene prediction method: GeneMarkS+.|hypothetical protein	JMA_3133(	6.385	23.135	10.545	125.375	367.815	213.845	110.415	320.13	189.43
JMA_RS06960	Derived by automated computational analysis using gene prediction method: Protein Homology.|hypothetical protein	JMA_1398(	160.07	658.915	300.605	5105.33	16991.94	9805.79	4494.85	14786.68	8672.395
JMA_RS11550	Catalyzes the reversible oxidation of malate to oxaloacetate {ECO:0000255|HAMAP-Rule:MF_00487}|(S)-malate + NAD(+) = oxaloacetate + NADH {ECO:0000255|HAMAP-Rule:MF_00487}|malate dehydrogenase|Malate dehydrogenase {ECO:0000255|HAMAP-Rule:MF_00487}	JMA_2366(	1364.145	5659.79	4104.4	19963.06	67336.28	62449.27	17574.67	58639.075	55370.295
JMA_RS11955	Derived by automated computational analysis using gene prediction method: Protein Homology.|hypothetical protein|Uncharacterized protein YtzB	JMA_2447(	451.36	1939.39	409.395	6082.685	21182.305	5594.045	5356.96	18435.84	4933.72
JMA_RS10745	Derived by automated computational analysis using gene prediction method: Protein Homology.|transcriptional regulator	JAM_2196(	188.79	815.305	526.61	2381.2	8344.845	6877.645	2095.94	7264.975	6091.69
JMA_RS11555	Isocitrate + NADP(+) = 2-oxoglutarate + CO(2) + NADPH|isocitrate dehydrogenase|Isocitrate dehydrogenase [NADP]	JMA_2367(	1235.13	5620.7	3619.54	18370.62	67917.8	56077.925	16171.745	59140.165	49742.065
JMA_RS10560	Derived by automated computational analysis using gene prediction method: Protein Homology.|hypothetical protein	JMA_2157(	165.56	822.33	544.015	537.375	2169.25	1872	473.055	1887.195	1657.39
JMA_RS01145	Involved in copper export {ECO:0000269|PubMed:12876283, ECO:0000269|PubMed:22663904}|ATP + H(2)O + Cu(2+)(Side 1) = ADP + phosphate + Cu(2+)(Side 2)|ATPase|Copper-exporting P-type ATPase B	JMA_0198(	42.73	218.24	109.625	142.09	590.02	382.415	125.075	513.375	338.085
JMA_RS20185	Derived by automated computational analysis using gene prediction method: Protein Homology.|hypothetical protein	JMA_4133(	0.86	5.35	4.755	6.13	30.885	35.935	5.395	26.87	31.915
JMA_RS12660	Derived by automated computational analysis using gene prediction method: Protein Homology.|branched-chain amino acid transporter II carrier protein|Branched chain amino acid transport system carrier protein BrnQ|Component of the transport system for branched-chain amino acids (leucine, isoleucine and valine) Which is coupled to a proton motive force {ECO:0000250}	JMA_2568(	829.22	5766.025	2697.75	2362.145	13390.86	8227.16	2079.835	11654.705	7288.995

JMA_RS12635	Derived by automated computational analysis using gene prediction method: Protein Homology.|sporulation protein|Sporulation protein YunB|Required for sporulation {ECO:0000269|PubMed:12662922}	JMA_2563	9.84	82.03	49.445	9.505	64.835	51.195	8.365	56.41	45.17
JMA_RS05210	Derived by automated computational analysis using gene prediction method: GeneMarkS+.|hypothetical protein	JMA_1050	16.065	135.2	77.98	129.25	881.17	652.925	113.785	766.765	578.245
JMA_RS12060	Derived by automated computational analysis using gene prediction method: Protein Homology.|hypothetical protein|Uncharacterized protein YwcH	JMA_2470	11.51	100.575	26.745	245.715	1736.635	582.645	216.27	1511.345	514.92
JMA_RS05660	Catalyzes the irreversible transfer of a propylamine group from the amino donor S-adenosylmethioninamine (decarboxy- AdoMet) to putrescine (1,4-diaminobutane) to yield spermidine {ECO:0000255|HAMAP-Rule:MF_00198}|S-adenosyl 3-(methylthio)propylamine + putrescine = 5'-S-methyl-5'-thioadenosine + spermidine {ECO:0000255|HAMAP-Rule:MF_00198}|spermidine synthase|Polyamine aminopropyltransferase {ECO:0000255|HAMAP-Rule:MF_00198}	JMA_1336	343.68	3410.435	378.125	6066.37	48754.015	6674.86	5340.335	42434.265	5872.43
JMA_RS07820	[illegible] germination in response to muropeptides, signaling bacteria to exit dormancy PrkC is thus a germination receptor that binds peptidoglycan fragments containing m-Dpm (meso-diaminopimelate), which act as spore germinants Probably autophosphorylates and phosphorylates FusA (EF-G, elongation factor G); the latter modification is likely necessary for germination in response to peptidoglycan {ECO:0000269|PubMed:18984160}|ATP + a protein = ADP + a phosphoprotein|serine/threonine protein kinase|Serine/threonine-protein kinase PrkC	JMA_1577	1171.675	14532.7	1979.37	5893.145	59489.015	10223.005	5188.315	51793.075	9020.085
JMA_RS08025	Derived by automated computational analysis using gene prediction method: Protein Homology.|flagellar biosynthesis protein FlgB|Flagellar basal body rod protein FlgB|Structural component of flagellum, the bacterial motility apparatus Part of the rod structure of flagellar basal body {ECO:0000269|PubMed:9159525}	JMA_1619	28.905	372.17	57.04	1156.16	11957.03	2257.875	1018.785	10394.39	1982.295
JMA_RS09850	Derived by automated computational analysis using gene prediction method: Protein Homology.|hypothetical protein|Uncharacterized protein YqjA	JMA_2006	1.385	17.99	10.445	10.99	116.62	86.045	9.68	101.555	76.11
JMA_RS03020	glutamate to 2-oxoglutarate and ammonia, thereby playing a key role at the intersection of the carbon and nitrogen metabolic pathways Is strictly specific for NAD(+)/NADH as the acceptor/donor, since it cannot use NADP(+)/NADPH May function in vivo in the catabolic direction Also catalyzes at low rates the oxidative deamination of L-norvaline, L-2-aminobutyrate, L-valine and L-isoleucine, and the reductive amination of 2-oxovalerate and 2-oxobutyrate {ECO:0000269|PubMed:23508687}|L-glutamate + H(2)O + NAD(+) = 2-oxoglutarate + NH(3) + NADH {ECO:0000269|PubMed:23508687}|glutamate dehydrogenase|NAD(+)-dependent glutamate dehydrogenase {ECO:0000303|PubMed:23508687}	JMA_0595	15.39	201.275	63.85	155.97	1658.26	668.675	137.435	1443.605	591.56
JMA_RS20840	DNA polymerase III is a complex, multichain enzyme responsible for most of the replicative synthesis in bacteria This DNA polymerase also exhibits 3' to 5' exonuclease activity The alpha chain is the DNA polymerase (By similarity) {ECO:0000250}|Deoxynucleoside triphosphate + DNA(n) = diphosphate + DNA(n+1)|hypothetical protein|DNA polymerase III subunit alpha	JMA_0490	0.08	1.06	1.935	0.315	3.465	7.865	0.275	3.015	6.92
JMA_RS10310	[illegible] diphosphate into isopentenyl diphosphate (IPP) and dimethylallyl diphosphate (DMAPP) {ECO:0000255|HAMAP-Rule:MF_00191}|Dimethylallyl diphosphate + NAD(P)(+) + H(2)O = (E)-4-hydroxy-3-methylbut-2-en-1-yl diphosphate + NAD(P)H {ECO:0000255|HAMAP-Rule:MF_00191}|4-hydroxy-3-methylbut-2-enyl diphosphate reductase|4-hydroxy-3-methylbut-2-enyl diphosphate reductase {ECO:0000255|HAMAP-Rule:MF_00191}	JMA_2107	2.815	38.705	12.28	23.205	258.225	104.69	20.42	224.665	92.61
JMA_RS07415	Derived by automated computational analysis using gene prediction method: Protein Homology.|protoheme IX farnesyltransferase|Protoheme IX farnesyltransferase 2 {ECO:0000255|HAMAP-Rule:MF_00154}|Converts heme B (protoheme IX) to heme O by substitution of the vinyl group on carbon 2 of heme B porphyrin ring with a hydroxyethyl farnesyl side group {ECO:0000255|HAMAP- Rule:MF_00154}	JMA_1494	193.425	2687.08	524.275	727.99	8226.855	2047	640.66	7161.29	1807.675
JMA_RS13595	Derived by automated computational analysis using gene prediction method: Protein Homology.|hypothetical protein	JMA_2768	375.695	5515.665	3275.66	3023.18	35939.835	27383.87	2661.345	31272.375	24244.715

JMA_RS01220	Part of the ABC transporter complex PhnCDE involved in phosphonates import Responsible for energy coupling to the transport system {ECO:0000255|HAMAP-Rule:MF_01713}|ATP + H(2)O + phosphonate(Out) = ADP + phosphate + phosphonate(In) {ECO:0000255|HAMAP-Rule:MF_01713}|ABC transporter ATP-binding protein|Phosphonates import ATP-binding protein PhnC {ECO:0000255|HAMAP-Rule:MF_01713}	JMA_2298(	6.525	114.085	24.925	74.74	1058.695	288.33	65.84	921.335	254.035
JMA_RS06235	Converts sucrose in nectar to glucose and fructose|Hydrolysis of terminal, non-reducing (1->4)-linked alpha-D-glucose residues with release of alpha-D-glucose|oligo-1,6-glucosidase|Alpha-glucosidase	JMA_1265(	75.465	1434.01	325.275	915.445	14038.61	3997.865	806.1	12210.335	3525.09
JMA_RS12100	Derived by automated computational analysis using gene prediction method: Protein Homology.|hypothetical protein|Uncharacterized protein YtmB	JMA_2479(	1.695	33.675	11.585	13.47	218.015	96.95	11.875	189.805	86.005
JMA_RS05485	involved in the sodium dependent uptake of proline; Derived by automated computational analysis using gene prediction method: Protein Homology.|proline:sodium symporter PutP|Osmoregulated proline transporter|Catalyzes the sodium-dependent uptake of extracellular proline	JMA_1108(	5.86	117.715	25.055	90.825	1473.525	392.545	79.985	1281.835	346.055
JMA_RS08015	Clp protease ATPase|ATP-dependent protease ATPase subunit HslU1	JMA_1617(	11.83	243.33	43.1	172.55	2870.27	627.82	151.985	2497.305	552.015
JMA_RS15970	ribosomal N-acetyltransferase YdaF|Uncharacterized N-acetyltransferase YoaA	JMA_3261(	2.285	47.535	14.01	3.555	60.6	23.665	3.135	52.72	20.935
JMA_RS12430	MFS transporter|Uncharacterized MFS-type transporter YuxJ	JMA_2546(	44.44	942.215	215.08	491.605	8427.525	2412.965	432.805	7332.21	2127.845
JMA_RS08005	Tyrosine recombinase XerD {ECO:0000255|HAMAP-Rule:MF_01807}|Site-specific tyrosine recombinase, which acts by catalyzing the cutting and rejoining of the recombining DNA molecules The XerC-XerD complex is essential to convert dimers of the bacterial chromosome into monomers to permit their segregation at cell division It also contributes to the segregational stability of plasmids {ECO:0000255|HAMAP-Rule:MF_01807}	JMA_1615(	86.435	1845.625	163.725	1321.77	22863.42	2447.955	1164.625	19896.41	2142.525
JMA_RS12090	Catalyzes the formation of S-adenosylmethionine from methionine and ATP The overall synthetic reaction is composed of two sequential steps, AdoMet formation and the subsequent tripolyphosphate hydrolysis which occurs prior to release of AdoMet from the enzyme {ECO:0000255|HAMAP-Rule:MF_00086}|ATP + L-methionine + H(2)O = phosphate + diphosphate + S-adenosyl-L-methionine {ECO:0000255|HAMAP-Rule:MF_00086}|S-adenosylmethionine synthetase|S-adenosylmethionine synthase {ECO:0000255|HAMAP-Rule:MF_00086}	JMA_2477(	1.57	35.255	11.655	9.235	168.65	71.33	8.135	146.74	63.07
JMA_RS18610	Derived by automated computational analysis using gene prediction method: GeneMarkS+.|hypothetical protein	JMA_0067(	0.405	9.265	2.1	0.63	11.54	3.595	0.55	10.05	3.19
JMA_RS08000	Catalyzes the folate-dependent formation of 5-methyl-uridine at position 54 (M-5-U54) in all tRNAs {ECO:0000255|HAMAP- Rule:MF_01037}|5,10-methylenetetrahydrofolate + uracil(54) in tRNA + FADH(2) = tetrahydrofolate + 5-methyluracil(54) in tRNA + FAD {ECO:0000255|HAMAP-Rule:MF_01037}|tRNA (uracil-5-)-methyltransferase|Methylenetetrahydrofolate--tRNA-(uracil-5-)-methyltransferase TrmFO {ECO:0000255|HAMAP-Rule:MF_01037}	JMA_1614(	284.23	6793.22	526.77	1395.34	27140.36	2578.12	1228.65	23627.32	2262.86
JMA_RS04420	D-glycerate + NAD(P)(+) = 2-hydroxy-3-oxopropanoate + NAD(P)H|tartronate semialdehyde reductase|2-hydroxy-3-oxopropionate reductase	JMA_0876(	53.065	1289.35	458.925	702.355	13832.59	6224.33	618.66	12038.865	5501.015
JMA_RS05130	Catalyzes the transfer of endogenously produced octanoic acid from octanoyl-acyl-carrier-protein onto the lipoyl domain of GcvH, an intermediate carrier during protein lipoylation {ECO:0000255|HAMAP-Rule:MF_02118}|Octanoyl-[acyl-carrier-protein] + protein = protein N(6)-(octanoyl)lysine + [acyl-carrier-protein] {ECO:0000255|HAMAP-Rule:MF_02118}|octanoyltransferase|Octanoyltransferase LipM {ECO:0000255|HAMAP-Rule:MF_02118}	JMA_1032(	32.855	876.325	96.505	267.875	5794.545	783.75	236.04	5042.05	688.19
JMA_RS08010	heat shock protein involved in degradation of misfolded proteins; Derived by automated computational analysis using gene prediction method: Protein Homology.|ATP-dependent protease subunit HslV|ATP-dependent protease subunit ClpQ|Protease subunit of a proteasome-like degradation complex {ECO:0000250}	JMA_1616(	10.69	293.85	27.085	104.61	2328.965	261.105	92.14	2026.51	228.84
JMA_RS02865	Derived by automated computational analysis using gene prediction method: Protein Homology.|hypothetical protein|Putative 23S rRNA (guanine-N(1)-)-methyltransferase YxjB	JMA_0561(	6.375	182.485	73.155	65.145	1510.6	769.915	57.375	1314.42	680.77
JMA_RS06505	Derived by automated computational analysis using gene prediction method: Protein Homology.|branched-chain amino acid ABC transporter permease|Uncharacterized membrane protein AF_1755	JMA_1306(	54.82	1603.415	137.88	949.915	22564	2359.845	836.43	19646.235	2070.44
JMA_RS04925	Derived by automated computational analysis using gene prediction method: Protein Homology.|RNA-binding protein	JMA_0984(	25.795	791.805	236.555	286.26	7132.55	2702.915	252.08	6209	2390.49

JMA_RS12105	Derived by automated computational analysis using<br>gene prediction method: Protein<br>Homology.|peptidase|Uncharacterized peptidase YtmA	JMA_2480	1.46	50.81	8.565	10.675	302.515	64.08	9.405	263.335	56.53
JMA_RS06045	Derived by automated computational analysis using<br>gene prediction method: Protein Homology.|spore<br>protein	JMA_1221	74.5	2698.895	195.925	829.76	24358.83	2140.825	730.665	21199.32	1875.03
JMA_RS12095	Involved in the gluconeogenesis Catalyzes the<br>conversion of oxaloacetate (OAA) to<br>phosphoenolpyruvate (PEP) through direct phosphoryl<br>transfer between the nucleoside triphosphate and OAA<br>{ECO:0000255|HAMAP-Rule:MF_00453}|ATP +<br>oxaloacetate = ADP + phosphoenolpyruvate + CO(2)<br>{ECO:0000255|HAMAP-<br>Rule:MF_00453}|phosphoenolpyruvate<br>carboxykinase|Phosphoenolpyruvate carboxykinase<br>[ATP] {ECO:0000255|HAMAP-Rule:MF_00453}	JMA_2478	0.95	35.325	8.225	7.75	234.245	69.97	6.82	203.925	61.99
JMA_RS02850	Part of the ABC transporter complex FhuBGCD<br>involved in iron(3+)-hydroxamate import Responsible<br>for energy coupling to the transport system (By<br>similarity) {ECO:0000250}|ATP + H(2)O + iron<br>chelate(Out) = ADP + phosphate + iron chelate(In)|iron-<br>enterobactin transporter ATP-binding protein|Iron(3+)-<br>hydroxamate import ATP-binding protein FhuC	JMA_0558	2.67	99.9	36.3	27.795	844.345	387.24	24.48	734.88	342.035
JMA_RS09005	Catalyzes the formation of 6,7-dimethyl-8-<br>ribityllumazine by condensation of 5-amino-6-(D-<br>ribitylamino)uracil with 3,4-dihydroxy-2-butanone 4-<br>phosphate This is the penultimate step in the<br>biosynthesis of riboflavin {ECO:0000255|HAMAP-<br>Rule:MF_00178}|1-deoxy-L-glycero-tetrulose 4-<br>phosphate + 5- amino-6-(D-ribitylamino)uracil = 6,7-<br>dimethyl-8-(D- ribityl)lumazine + 2 H(2)O +<br>phosphate {ECO:0000255|HAMAP-<br>Rule:MF_00178}|6,7-dimethyl-8-ribityllumazine<br>synthase|6,7-dimethyl-8-ribityllumazine synthase<br>{ECO:0000255|HAMAP-Rule:MF_00178}	JMA_1818	147.985	5683.195	506.785	132.63	4156.585	506.135	116.69	3619.605	448.155
JMA_RS17160	important biological processes such as cell separation,<br>cell-wall turnover, competence for genetic<br>transformation, formation of the flagella - in particular<br>of its basal body - and sporulation Has a high affinity<br>for teichoic acid-endowed peptidoglycan LytC is<br>required for efficient swarming motility but not at the<br>level of cell separation or flagellum biosynthesis<br>Rather, LytC appears to be important for proper<br>flagellar function {ECO:0000269|PubMed:1682302,<br>ECO:0000269|PubMed:19542270}|Hydrolyzes the link<br>between N-acetylmuramoyl residues and L-amino acid<br>residues in certain cell-wall glycopeptides<br>{ECO:0000269|PubMed:1682302}|hypothetical<br>protein|N-acetylmuramoyl-L-alanine amidase LytC	JMA_0367	0.955	36.98	14.13	10.63	332.725	160.095	9.36	289.59	141.35
JMA_RS15210	Derived by automated computational analysis using<br>gene prediction method: Protein Homology.|alpha/beta<br>hydrolase|AB hydrolase superfamily protein YisY	JMA_3101	21.37	873.7	236.855	259.995	8612.965	2935.565	229	7495.615	2591.085
JMA_RS05410	[illegible]<br>formyltetrahydrofolate to 5-phospho-ribosyl-<br>glycinamide (GAR), producing 5-phospho-ribosyl-N-<br>formylglycinamide (FGAR) and tetrahydrofolate<br>{ECO:0000255|HAMAP-Rule:MF_01930}|10-<br>formyltetrahydrofolate + N(1)-(5-phospho-D-<br>ribosyl)glycinamide = tetrahydrofolate + N(2)-formyl-<br>N(1)-(5- phospho-D-ribosyl)glycinamide<br>{ECO:0000255|HAMAP-<br>Rule:MF_01930}|phosphoribosylglycinamide<br>formyltransferase|Phosphoribosylglycinamide<br>formyltransferase {ECO:0000255|HAMAP-<br>Rule:MF_01930}	JMA_1091	23.36	983.44	90.055	336.265	11467.425	1278.505	296.065	9980.61	1121.275
JMA_RS04425	Derived by automated computational analysis using<br>gene prediction method: Protein<br>Homology.|hydroxyacid dehydrogenase	JMA_0877	11.02	480.73	70.895	88.795	3131.325	568.565	78.11	2724.505	499.435
JMA_RS02855	2 reduced ferredoxin + NADP(+) + H(+) = 2 oxidized<br>ferredoxin + NADPH {ECO:0000255|HAMAP-<br>Rule:MF_01685}|thioredoxin reductase|Ferredoxin--<br>NADP reductase 1 {ECO:0000255|HAMAP-<br>Rule:MF_01685}	JMA_0559	3.3	163.47	47.645	33.52	1344.04	491.88	29.495	1169.675	433.95
JMA_RS06035	Derived by automated computational analysis using<br>gene prediction method: Protein Homology.|DNA<br>glycosylase|A/G-specific adenine DNA<br>glycosylase|Adenine glycosylase active on G-A<br>mispairs Has glycosylase and nicking activities and is<br>active at A/G and A/GO sites<br>{ECO:0000269|PubMed:9737967}	JMA_1219	24.115	1200.31	261.455	217.915	8782.115	2392.395	191.86	7641.53	2108.285
JMA_RS03025	Subtilisin is an extracellular alkaline serine protease, it<br>catalyzes the hydrolysis of proteins and peptide<br>amides|Hydrolysis of proteins with broad specificity<br>for peptide bonds, and a preference for a large<br>uncharged residue in P1 Hydrolyzes peptide<br>amides|peptidase S8|Subtilisin	JMA_0596	3.89	220.795	38.985	33.115	1530.115	336.675	29.16	1332.34	296.72

JMA_RS04930	Derived by automated computational analysis using gene prediction method: Protein Homology.|cation transporter|Cadmium, cobalt and zinc/H(+)-K(+) antiporter|Involved in divalent cation and potassium homeostasis in the cell Catalyzes the active efflux of zinc, cadmium and cobalt, in exchange for potassium and H(+) ions (By similarity) {ECO:0000250}	JMA_0985(	4.89	319.44	67.14	38.34	2033.645	539.74	33.775	1770.03	476.575
JMA_RS04935	Derived by automated computational analysis using gene prediction method: Protein Homology.|ArsR family transcriptional regulator|HTH-type transcriptional repressor CzrA|Metal-responsive transcriptional regulator that represses transcription of cadA and the czcD-trkA operon by binding specifically to their promoter Binding of zinc causes the repressor to dissociate from the DNA {ECO:0000269|PubMed:16430705}	JMA_0986(	4.635	346.66	56.075	38.025	2312.925	469.77	33.46	2013.95	414.585
JMA_RS03820	Derived by automated computational analysis using gene prediction method: Protein Homology.|cell cycle protein|Rod shape-determining protein RodA|Required for the maintenance of the rod cell shape Required for the expression of the enzymatic activity of the penicillin-binding protein 2 (PBP2) (By similarity) {ECO:0000250}	JMA_0764(	16.755	1344.94	107.26	129.3	8427.28	812.435	113.945	7334.07	711.27
JMA_RS15100	Derived by automated computational analysis using gene prediction method: Protein Homology.|acyl-CoA dehydrogenase|Putative acyl-CoA dehydrogenase YdbM	JMA_3080(	9.765	1003.755	88.14	60.73	5065.175	544.3	53.48	4407.995	477.59
JMA_RS03815	Derived by automated computational analysis using gene prediction method: Protein Homology.|hypothetical protein|Putative lipid kinase BmrU|May catalyze the ATP-dependent phosphorylation of lipids other than diacylglycerol (DAG) In fact, is not able to exhibit diacylglycerol kinase activity in vitro	JMA_0763(	13.68	1499.905	105.355	89.925	8006.84	679.435	79.21	6968.75	594.79
JMA_RS03810	Derived by automated computational analysis using gene prediction method: Protein Homology.|hypothetical protein|Uncharacterized protein MK0525	JMA_0762(	13.05	1873.3	132.865	109.655	12763.985	1092.875	96.555	11108.865	956.62
JMA_RS09025	(S)-dihydroorotate + H(2)O = N-carbamoyl-L-aspartate {ECO:0000255|HAMAP-Rule:MF_00220}|amidohydrolase|Dihydroorotase {ECO:0000255|HAMAP-Rule:MF_00220}	JMA_1823(	35.095	7483.275	627.04	507.775	87875.115	8988.95	447.195	76499.72	7890.575
JMA_RS09020	Converts 2,5-diamino-6-(ribosylamino)-4(3h)-pyrimidinone 5'-phosphate into 5-amino-6-(ribosylamino)-2,4(1h,3h)- pyrimidinedione 5'-phosphate|5-amino-6-(5-phospho-D-ribitylamino)uracil + NADP(+) = 5-amino-6-(5-phospho-D-ribosylamino)uracil + NADPH|5-amino-6-(5-phosphoribosylamino)uracilreductase|Riboflavin biosynthesis protein RibD	JMA_1822(	17.685	4552.78	492.42	223.73	46738.995	6212.525	196.975	40689.875	5460.82
JMA_RS09015	Catalyzes the dismutation of two molecules of 6,7-dimethyl-8-ribityllumazine, resulting in the formation of riboflavin and 5-amino-6-(D-ribitylamino)uracil {ECO:0000250}|2 6,7-dimethyl-8-(1-D-ribityl)lumazine = riboflavin + 4-(1-D-ribitylamino)-5-amino-2,6-dihydroxypyrimidine|riboflavin synthase subunit alpha|Riboflavin synthase	JMA_1821(	14.555	3993.15	431.15	129.84	28967.425	3857.535	114.29	25224.545	3393.265
